# Supplementary material for: Translation of culturally and contextually informed diabetes training for Aboriginal primary health care providers on Aboriginal client outcomes: Protocol of a cluster randomized crossover trial of effectiveness
Source: PLoS One. 2024 Jul 23;19(7):e0305472. doi: 10.1371/journal.pone.0305472 (PMC11265707; doi:10.1371/journal.pone.0305472)
Supplement: S2 File — (DOCX) [file pone.0305472.s002.docx]

| **PARTICIPANT INFORMATION SHEET**  **Training Program** | |
| --- | --- |
| **Project Title:** | **Aboriginal Diabetes Workforce Study** |

**You will be given a copy of this Participant Information and Consent Form to keep.**

**What is the purpose of this research?** Aboriginal Health Workers/Practitioners (AHW/P) and multidisciplinary health care providers have been involved in designing a diabetes training program for the primary health care workforce. The purpose of this study is to deliver and evaluate this culturally and contextually informed diabetes workforce training program. The evaluation will seek to understand the effect of the training program on knowledge, confidence, attitude, skills, and practice of Aboriginal Primary Health Care Service providers in providing care to clients with diabetes.

**Who can participate?**

- Aboriginal Health Worker/Practitioners who have completed or are currently enrolled in certificate III/IV in Aboriginal Primary Health Care
- Enrolled and registered nurse, allied health professionals, medical doctors.
- Who are current employees of a participating South Australian Aboriginal primary health care service
- Provide diabetes care to Aboriginal clients.

**Who is involved in this research?** All Aboriginal primary health care services across South Australia have been invited to participate in this research. The research is led by Associate Professor Odette Pearson, Population Health Platform Lead at Wardliparingga Aboriginal Health Equity at the South Australian Health and Medical Research Institute (SAHMRI) with research investigators from research, health, and education organisations. Your service is participating in this research and has agreed to allow AHW/P to participate during work hours in the Peer Support Network, for all participants to access a work computer and the internet to complete the training, and to participate in the on-site support provided by the Diabetes Educator employed by this study.

**What does this research study involve?** The research involves participating in the training program and the evaluation of the training program. The Training Program consists of three (3) components:

1. A state-wide **AHW/P Peer Support Network (PSN)**, consisting of monthly online sessions with AHW/P across South Australia, facilitated by a Credentialled Diabetes Educator (CDE). This network is for AHW/P only.
2. Diabetes educational content delivered through nine (9) self-paced **e-Learning modules**. The modules cover a variety of diabetes related topics such as healthy living, glucose monitoring, medicines and insulin, diabetes related complications and support for self-management.
3. **Onsite Support** **(OS)** that is facilitated by a Credentialled Diabetes Educator (CDE) at the service you work in.

**What will your participation involve?**

***Peer Support Network (AHW/P only)***

- Monthly involvement for up to 2 hours for 17 months (up to 17 times). This will involve listening and contributing to the discussions with your peers.

***e-Learning Modules***

- Complete nine e-Learning modules over 10 weeks. Each module takes approximately 1 hour to complete. A link will be sent to you by email. The modules are self-paced so you can do more than one per week if you choose to. You will receive 10 CDP and a certificate on completion of all modules.

***Onsite Support (OS)***

- This will include at least two onsite support sessions with the OS Facilitator. At the beginning of these sessions, participants and the OS Facilitator will develop a plan on what to cover in the OS sessions and agree on the number of sessions. These will be group sessions, if more than one staff member of the same service is participating in the project, and the content may align with what you are learning in the e-Learning modules. Half hour of the session will be dedicated to AHW/P only. The length of each session will be determined by you (& your colleagues) and the facilitator.

Your participation in this study is entirely voluntary. If you choose not to participate, this will not affect your relationship with your employer, the project team or the stakeholders involved in this project.

**What information will be collected from you?** To evaluate the effectiveness of the Aboriginal Informed Diabetes Training Program, you will be asked to complete several online surveys using RedCap, data capture program, before, during and after your participation in the research training program. Secure RedCap Survey Links will be sent to you by email. You will be followed up, up to 3 times by email and one by phone to complete the survey, but there are no consequences if you do not complete the survey. However, by doing so, you will help the researchers to know what did or did not work in the training.

The timing and description of the online surveys are as follows:

***At the start of the project (15 minutes)***

- A survey to collect your demographic information (5 mins)
- A survey that assesses your diabetes knowledge, practice, attitude, confidence, skills and practice (10 mins)

***After each Peer Support Network meeting (7 minutes x17)***

- An anonymous survey at the end of each session that will be used to improve the network. This is for AHW/Ps only

***Before the start of the e-Learning modules (10 minutes)***

- A survey that assesses your diabetes knowledge, practice, attitude, confidence, skills, and practice (10 mins)

***6 and 12 months after the second group*** *have completed the online learning modules (10 minutes)*

- A survey that assesses your diabetes **knowledge, practice, attitude, confidence, skills**, and **practice** (10 mins x2)

***Semi-structured interview (60 minutes)***

After you have completed the online modules, you will be invited to participate in an interview with a member of the research team, to explore your experience of the Training program. You do not have to participate in the interview. Individual responses will not be shared with your employer, the PSN or OS Facilitators, or other participants. You will be asked for permission to record the interview for translation and be given an opportunity to review your transcript to add or remove information.

**How will the researchers use the results?**

To protect anonymity of individual survey and interview results, only combined results will be reported. The results will be used to determine the effectiveness of the training program in supporting the Aboriginal primary health care workforce in managing the care of clients with diabetes. Furthermore, the results will be used to inform improvements in the existing training program. Only combined results will be shared with participants, primary care services and the state and national community-controlled health sector, registered training organisations, relevant workforce associations, diabetes stakeholder groups, and in published academic papers. You will not be identified in the sharing of results.

**What are the benefits and risks to you in participating?**

The Aboriginal Diabetes Workforce Training Program will equip you with additional knowledge on diabetes and its management and support you to deliver best practice diabetes care to your clients with diabetes. There is minimal risk associated with participating in the study.

**Withdrawing from the study**

You may choose to withdraw your consent to participate in the trial at any time by contacting a member of the research team, whose details are at the end of this information sheet. If you chose to withdraw your consent before the data analysis stage of the trial, your data will be separated and will not become part of the study findings. If you choose to withdraw after the data analysis, it will not be possible to retract your data. Withdrawing from the research will not affect your relationship with your employer or the research team. At any time throughout the trial if you would like to discuss your thoughts or concerns, the research team will be able to arrange appropriate support.

**Confidentiality & privacy**

There are strict processes in place to protect your privacy:

- Any information you provide to us will be de-identified and accessible only by the research team
- All data will be stored electronically on a password protected server at SAHMRI in accordance with data management policies and destroyed after 7 years.
- No third parties will be given access to the data
- The information you provide will only be used for the purposes of the study and no other, without your expressed permission

**Ethical Approval**

This Research Project was funded by the Medical Research Future Fund and has been assessed and approved by the *Aboriginal Health Research Ethics Committee* of the *Aboriginal Health Council of South Australia* and the *SA Department for Health and Wellbeing Human Research Ethics Committee.*

If you have any concerns or complaints regarding the ethical conduct of the research, please contact the Manager, Research and Ethics at the *Aboriginal Health Research Ethics Committee*, *Aboriginal Health Council of South Australia*, on 08 8273 7200 or email [research@ahcsa.org.au](mailto:research@ahcsa.org.au) or the HREC Executive Officer at the *SA Department for Health and Wellbeing Human Research Ethics Committee* on 08 82226 8102 or email [healthhumanresearchethicscommittee@sa.gov.au](mailto:healthhumanresearchethicscommittee@sa.gov.au)

**To discuss the study in more detail and flag your interest** please contact Associate Professor Odette Pearson, Population Health Platform Lead at Wardliparingga Aboriginal Health Equity, SAHMRI, on [Odette.Pearson@sahmri.com](mailto:Odette.Pearson@sahmri.com), Sana Ishaque, [Sana.Ishaque@sahmri.com](mailto:Sana.Ishaque@sahmri.com) or Tinarra Toohey, Tinarra.Toohey@sahmri.com or phone 08 81284000.
