# Supplementary material for: Translation of culturally and contextually informed diabetes training for Aboriginal primary health care providers on Aboriginal client outcomes: Protocol of a cluster randomized crossover trial of effectiveness
Source: PLoS One. 2024 Jul 23;19(7):e0305472. doi: 10.1371/journal.pone.0305472 (PMC11265707; doi:10.1371/journal.pone.0305472)
Supplement: S3 File — (DOCX) [file pone.0305472.s003.docx]

| **CONSENT FORM**  ***Training Program*** | |
| --- | --- |
| **Project Title:** | **Aboriginal Diabetes Workforce Study** |

- I have received information about this research project and the research project has been explained to me.
- I understand that the service I am employed by will provide access to a computer, the Internet, and supports my participation in the training during work hours.
- I have had a chance to ask questions and am comfortable with the answers I have been given.
- I understand what the research project is about and what I am being asked to do.
- I have volunteered to participate in the research project (THIS MEANS YOU CAN SAY NO).
- I understand that I do not have to participate in all data collection.
- I give permission to be followed up about an interview on the enablers and barriers of the training program and understand I do not have to consent to do an interview.
- I understand that I may withdraw from the research at any time without any negative impact from the research team or my employer. I do not have to give a reason.
- I understand that the research findings might be published in research reports, conferences or community reports/newsletters.
- I will not be identified in any published research findings and my personal information will remain confidential.
- I understand that I own my information (intellectual property) that I provide in the study.
- Should a distressing event occur while participation in the study, I give permission for a research team member to check on my welfare.

**Name of participant:** ­­­­­­­­­­­­­­­­­­­­­­­­­­­­­_______________________________________________________________

**Email address:** _____________________________________________________________________

**Signed:**  ________________________________ **Date:**  ______________________________

**I have explained the research project to the participant and believe that they understand what is involved.**

**Researcher’s name:** ­­­­­­­­­­­­­­­­­­­­­­­­­­­­­_____________________________________________________________

**Researcher’s signature:** ____________________________ **Date:** ___________________________

**Please send signed consent for to:** [[**sana.ishaque@sahmri.com**](mailto:sana.ishaque@sahmri.com)](mailto:sana.ishaque@sahmri.com) Or [**Tinarra.toohey@sahmri.com**](mailto:Tinarra.toohey@sahmri.com)

If you would like more information, or have any concerns or complaints about this research project, you can speak to:

- Associate Professor Odette Pearson, Population Health Platform Lead, Wardliparingga Aboriginal Health Equity, South Australian Health and Medical Research Institute (SAHMRI) Ph: 08 8128 4000 Email: [odette.pearson@sahmri.com](mailto:odette.pearson@sahmri.com)
- Manager, Research and Ethics, Aboriginal Health Research Committee, Aboriginal Health Council of South Australia

Ph: 08 8273 7200 Email: [research@ahcsa.org.au](mailto:research@ahcsa.org.au)

- HREC Executive Officer, SA Department of Health and Wellbeing Human Research Ethics Committee Officer

Ph: 08 8226 8102 Email: [healthhumanresearchethicscommittee@sa.gov.au](mailto:healthhumanresearchethicscommittee@sa.gov.au)
