# Supplementary material for: Translation of culturally and contextually informed diabetes training for Aboriginal primary health care providers on Aboriginal client outcomes: Protocol of a cluster randomized crossover trial of effectiveness
Source: PLoS One. 2024 Jul 23;19(7):e0305472. doi: 10.1371/journal.pone.0305472 (PMC11265707; doi:10.1371/journal.pone.0305472)
Supplement: S4 File — (DOCX) [file pone.0305472.s004.docx]

Attachment 2


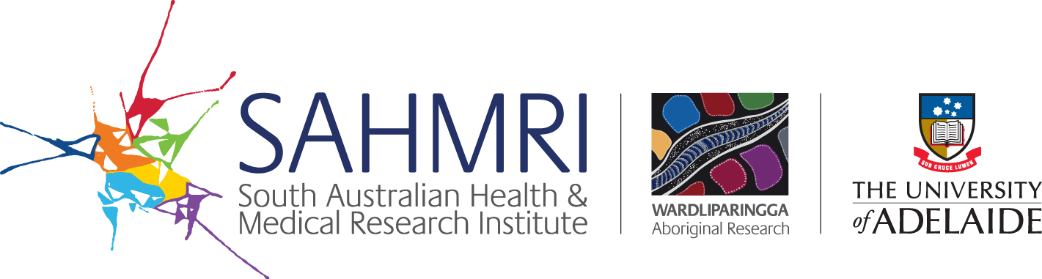


Aboriginal Informed Diabetes Training Program

PEER SUPPORT NETWORK

FACILITATOR GUIDE

[Inside front page]

This facilitator guide has been produced for *Translation of culturally informed diabetes training for Aboriginal Health Practitioners on Aboriginal patient outcomes: a cluster randomised trial of effectiveness* (APP1200314) funded by the Medical Research Future Fund.

The guide was compiled by the research team members Sana Isahque and Odette Pearson with input from research investigators Jane Giles and Natalie Wischer and colleagues Kate Colmer and Sarah Davey.

Contents

[Preface 3](#_bookmark0)

[Aboriginal Informed Diabetes Training Program 3](#_bookmark1)

[Aims of the diabetes training program 3](#_bookmark2)

[Peer Support Network - Facilitator Guide 3](#_bookmark3)

[Introduction 3](#_bookmark4)

[The purpose of this Network 4](#_bookmark5)

[Facilitator functions 4](#_bookmark6)

[Before the meeting 4](#_bookmark7)

[First Meeting (Session outline) 4](#_bookmark8)

[During the meeting (Session outline) 5](#_bookmark9)

[Ending the Meeting (last 20 minutes of the session) 5](#_bookmark10)

[After the meeting 5](#_bookmark11)

[Suggestions for facilitators 6](#_bookmark12)

[References 6](#_bookmark13)

[Attachments 6](#_bookmark14)

[Attachment 1 sample meeting agenda template 8](#_bookmark15)

[Attachment 2 Acknowledgment of Country 9](#_bookmark16)

[Attachment 3 Examples of Icebreaker activities 9](#_bookmark17)

[Attachment 4 Ground norms (to be repeated in the first 10 minutes of each session) 10](#_bookmark18)

[Attachment 5 Permission to Record 10](#_bookmark19)

[Attachment 6 Attendance Sheet 11](#_bookmark20)

[Attachment 7 Sample template to record meeting minutes 11](#_bookmark21)

[Attachment 8 Facilitator Reflection Form 12](#_bookmark22)

[Attachment 9 Topics to be discussed in the network aligned with the AHW/P professional scope of](#_bookmark23) [practice 13](#_bookmark23)

[Attachment 8 Activities/Strategies to cover the topics and Topic Template 15](#_bookmark24)

1. [Case study – template on how to present a case study 15](#_bookmark25)
2. [Stories/lived experiences – template 16](#_bookmark26)
3. [Guest speakers – contacting the guest speaker 16](#_bookmark27)
4. [Direct reflections template 16](#_bookmark28)

[Attachment 9 Tool kit Appendix 17](#_bookmark29)

1. [Evaluation survey (anonymous survey for the participants to complete at the end of each](#_bookmark30) [meeting) 17](#_bookmark30)
2. [Research team’s responsibilities 17](#_bookmark31)

# Preface

This document is prepared to guide the facilitator in coordinating the peer support sessions with Aboriginal Health Worker and Practitioner health professionals. It acknowledges that facilitators will have a depth and breadth of experience in facilitating a network of health professionals. The document is a guide only and is not meant to be prescriptive.

# Aboriginal Informed Diabetes Training Program

The Aboriginal Informed Diabetes Training Program has been developed in consultation with Aboriginal Community Controlled Health Organisations and South Australian government primary health care services. There have been multiple rounds of consultations that informed the design and conduct of the program.

There are three components of the training: peer support network, online modules, and onsite practice support. As part of the Diabetes Workforce project, the training program is to be delivered as part of a cluster randomised controlled trial across South Australia. The Project aims to provide additional knowledge and skills in diabetes management to Aboriginal health workers and practitioners (AHW/Ps) working with clients with diabetes and effectively bridge the gap between the coverage of the Cert III & IV and the scope of practice within the workplace regarding diabetes care.

## Aims of the diabetes training program

- To create a state-wide peer network of Aboriginal Health Workers and Practitioners working in chronic disease management to enable the sharing and learning of information and to support the translation of knowledge into practice
- To provide a minimum standard of knowledge of diabetes, related health complications and management across the Aboriginal Health Worker and Practitioner workforce in South Australia
- To increase knowledge of best practice management of diabetes and how it is applied within the primary health care setting and using the resources available within your community
- To promote knowledge translation through practical on-site support delivered within the local primary health care setting

# Peer Support Network - Facilitator Guide

## Introduction

The peer support network for the Aboriginal Informed Diabetes Training Program will run for two hours each month. All Aboriginal health workers/practitioners employed in a primary health care setting (AHW/Ps) working in chronic disease management across South Australia are invited to be members. A peer support group/network is a group of individuals with common experiences and concerns who meet to support each other professionally, emotionally, and otherwise (cite). The frequency and duration of this network will be open to revision as the network grows and evolves with feedback from participants.

The network will be facilitated by a diabetes nurse educator (DNE). The role of the DNE is to facilitate discussion and content of the meetings. The DNE will also be required to arrange for

resources: such as guest speakers, to guide the participants on specific ways of presenting their lived experiences/stories (e.g. case study, reflections, personal experience, etc).

A professional scope of practice is the complete range of activities and functions that a health care provider is educated, competent, and authorised to perform in their particular professional role. This facilitator guide has been developed with consideration of the professional scope of practice document for AHW/Ps in Queensland available from the Australian Indigenous Health*InfoNet* website. This document has been considered because the training/roles of the AHW/Ps described in the document are closely related to the training provided to them in South Australia. This model has been ascertained in consultation with the Aboriginal Health Council of South Australia Registered Training Organisation. The scope of practice is also determined by the organisation/agency a professional is employed by. This scope of practice is called individual scope of practice and is usually defined in the job description document. Since this network is run to enhance the confidence, knowledge, and skills of AHW/P and to support them in their job roles and responsibilities; the topics to be discussed in the network are aligned to the AHW/P professional scope of practice.

## The purpose of this Network

The purpose of the Network is to create a state-wide peer network of Aboriginal Health Workers and Practitioners working in chronic disease management to enable the sharing and learning of information and to support the translation of knowledge into practice. These will be achieved by providing opportunities to support and learn from each other informal and formal peer mentoring, improved relationships and social networks, personal growth in knowledge and confidence of participants, and improved feelings of being accepted as a professional.

This network is curriculum based with a focus on diabetes related education and the purpose of the network is to support the translation of the diabetes related content provided in the online modules into practice. However, the overall network's aim is broader than this.

From a facilitating point of view, it is important to remember that the sessions need to be open for discussion and not just be educational lecture-style webinars.

# Facilitator functions

## Before the meeting

- Develop a session agenda and email it to the members at least a week before the meeting. A sample meeting agenda is attached (Attachment 1)
- Ask participants if anyone would like any items to be included in the agenda
- Arrange for resources required to run the session on the planned agenda

## First Meeting (Session outline)

- Acknowledgement of Country (Attachment 2)
- One minute silence to remember those who have gone before us
- Welcome to members,
- Purpose of the Network
- Introductions & ice-breaker activity (Attachment 3); what do individuals want to get out of the Network?
- How the Network will run (email sent out to all members advising of upcoming meeting; members decide on topics to cover at next meeting, at the end of each network; review the running and outcomes of the network at the end of each meeting – complete a short anonymous survey that is emailed through a link)
- Discuss ground norms (Attachment 4) – is there anything that needs changing or adding?
- Talk about recording the sessions – is this something the group would like to do, asking permission to record at the beginning of each session (Attachment 5)
- Touch base re the participants’ wellbeing
- Record attendance (Attachment 6),– 30 minutes
- Online Teams site posting questions, will not be staffed daily, questions can be raised and these will be covered in the next session, resources available on the site, Network meetings held via Teams link

## During the meeting (Session outline)

- Acknowledgement of Country (Attachment 2), one-minute silence to remember those who have gone before us, welcome to members, make time for introductions & ice-breaker activity (Attachment 3), revision of ground norms (Attachment 4), permission to record (Attachment 5), touch base re participants’ wellbeing, and record attendance (Attachment 6),– 30 minutes
- Review/answer any questions from the last session – 10 minutes
- Topic coverage – 40 minutes
- Questions/open discussion (possible practices changes) 20 minutes
- Plan/discuss re next session – 20 minutes

## Ending the Meeting (last 20 minutes of the session)

Closing the meeting - it is preferred to close the meeting on time. However, if the group is caught up in an intense discussion, a few extra minutes may be allowed.

- Provide a short notification of approximately 20 minutes before the session is to finish
- Invite members to make any final comments
- Make mention of the literature or other material that has been used in the group or provided for reference
- Provide a summary of the meeting and any conclusions that the group agreed on
- Announce the date, topic, and time of the next meeting; with the help of members
- Conclude the meeting by appreciating the group for their attendance and participation. Encourage members to support one another in between the meetings
- Inform the participants that they will receive a link to an anonymous survey via email for collecting their feedback on the session and that this will ensure that future sessions can be improved if needed

## After the meeting

- Circulate the meeting minutes (Attachment 7) within a week after the session to all the participants and those who missed the meeting. Minutes are not verbatim and just cover the main topics discussed
- Document what needs to be done before the next meeting. Source resources, follow up questions that needed more information, make arrangements for the next meeting (e.g. guest speaker, case study)
- Decide if you think the meeting went well (record your own impression on the reflection form – Attachment 8)
- Decide if enough people attended, think of avenues for the promotion of the network

## Suggestions for facilitators

- The facilitator may start the conversation, but the members do not only respond to him/her but rather speak to each other. The discussion needs to happen in an open conversation style that follows a natural flow
- There needs to be constant encouragement to attend the sessions from facilitator in order to gain the benefits
- Facilitator addresses people by their name so that other members can learn each other’s name and get to do the same. This demonstrates that the facilitator cares and respects the participants
- Facilitator’s need to be aware of when to extend a discussion and when to move on to the next topic. When the members who are relatively quiet start a topic, the facilitator may need to spend more time and encourage discussion on the topic
- Be sure to notice and include members who may be quiet and less confident to participate appear withdrawn and bored in the session in discussion
- Listening is an important skill for the role of facilitator. It is important to know when to talk and when to let other members speak. Facilitator needs to be aware and comfortable with silent periods during the sessions and should not try to fill those up with over speaking
- Facilitator’s needs to be open to the group feedback and be prepared to take the suggestions on board to improve the network

# References

1. A practicle guide for sessting up a peer support group. NDIS; 2019, Available from: [https://www.limbs4life.org.au/.](https://www.limbs4life.org.au/)
2. Support group facilitation guide. Mental Health America’s Center for peer support; 2016.

Available from: <https://www.mhanational.org/resources-and-support-peers>.

1. Peer support facilitator guide. Health and Safety Directorate; 2020. Available from: <https://pubdocs.worldbank.org/en/178031585674457717/Peer-Group-Facilitator-Guide.pdf>
2. Using peer support in developing empowering meantal health services (UPSIDES): Background, rationale and methodology. Annals of Global Health; 2019, 85 (1), 1-10.
3. Melany Cueva, Regina Kuhnley, Laura J. Revels, Katie Cueva, Mark Dignan & Anne P. Lanier. Bridging storytelling traditions with digital technology. Int J. of Circumpolar Health. 2013; 72 (1).
4. Heart health action in Aboriginal Commuities: Translating training into practice, final evaluation report. Available from: [https://www.vaccho.org.au/assets/01-RESOURCES/TOPIC-](https://www.vaccho.org.au/assets/01-RESOURCES/TOPIC-AREA/NUTRITION/Health-Action-in-Aboriginal-Communities-eval-Report-Final-2009-09-18.pdf) [AREA/NUTRITION/Health-Action-in-Aboriginal-Communities-eval-Report-Final-2009-09-18.pdf](https://www.vaccho.org.au/assets/01-RESOURCES/TOPIC-AREA/NUTRITION/Health-Action-in-Aboriginal-Communities-eval-Report-Final-2009-09-18.pdf)

# Attachments

- 1. Sample meeting agenda template
  2. Acknowledgment of Country
  3. Examples of ice-breaker activities
  4. Ground norms
  5. Permission to record
  6. Attendance sheet
  7. Same template to record meeting minutes
  8. Facilitator reflection form – to be completed after each meeting
  9. Topics to be discussed in the network aligned with the AHW/Ps professional scope of practice
  10. Activities/Strategies to cover the topics and their templates
  11. Tool kit Appendix

## Attachment 1 sample meeting agenda template


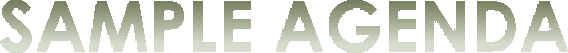

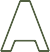

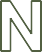


Aboriginal Informed Diabetes Training Program –

Peer Support Network

*Date* | Time, location *Zoom details:* Facilitator Name

| **Item** | **Description** | **Expected outcome** |
| --- | --- | --- |
| **Date** |  |  |
| **Time** | **The start and finish time of the meeting** |  |
| **Attendees &**  **Apologies** | **Who is present and who is not** |  |
| 1 | Acknowledgement of Country |  |
| 2 | - Welcome, - Introductions & ice-breaker activity - reminder of ground norms - permission to record - touch base participant wellbeing |  |
| 3 | Review/answer any questions from the last session (Minutes from the last meeting) |  |
| 4 | Topic coverage for the current session |  |
| 5 | Reports – things the group has been doing | Comments, Questions |
| 6 | Any other business |  |
| 7 | Planning for next meeting |  |

## Attachment 2 Acknowledgment of Country

The following is one example of an Acknowledgement of Country which can be used at the start of each session. This wording is for facilitation on Kaurna Country and will change depending on where the facilitator is located.

#### Adelaide plains region

I am on the lands of the Kaurna people. I acknowledge and respect the Kaurna people, the traditional custodians whose ancestral lands I am upon today. I also acknowledge and respect all the traditional custodians of the ancestral lands we are all on across South Australia. We acknowledge the deep feelings of attachment and relationship of the traditional custodians to their Place. We also pay respects to the cultural authority of Aboriginal and Torres Strait Islander peoples visiting/ attending from all areas of South Australia / Australia present online.

**For locations outside of Adelaide**, the below acknowledgement of Country can be used to replace the Kaurna traditional custodians with the relevant traditional custodians:

We acknowledge and respect the traditional custodians whose ancestral lands we are meeting upon here today, the people. We acknowledge the deep feelings of attachment and relationship of the people to their Country. We also pay respects to the cultural authority of Aboriginal and Torres Strait Islander people visiting/ attending from other areas of South Australia / Australia present here

## Attachment 3 Examples of Icebreaker activities

The icebreaker activities are helpful in bringing the group together, especially in the first few sessions.

- Everyone tells their name and two interesting facts about them
- Two truths and a lie- everyone tells two truths and a lie about themselves and others guess which one of the three is a lie
- The facilitator can perform any activity they think would be suitable as an icebreaker

## Attachment 4 Ground norms (to be repeated in the first 10 minutes of each session)

The ground norms need to be stated clearly at the start of each session, even if there are no new members present. **Consider distributing a copy of the ground norms to the members via email.**

The dot points below are only a starting point for developing this networks’ ground norms. It is suggested that the facilitator revise the group norms with the participants and make them group specific.

- Respect: It is of utmost importance that all members understand and maintain respect for each other and each other’s opinions at all times
- Listening without interrupting or jumping in
- Avoid any side stories and conversations during the meeting
- Avoid judging others
- Boundaries
- Confidentiality and its limitations: Only share what you personally feel comfortable sharing. The members are prohibited from disclosing anything that is shared in the meetings. This includes identifying the members who are present. Names, addresses, phone numbers, and emails shared must be kept confidential. Any personal stories or identifiable information shared in the group must not be shared outside without permission. There are certain limitations to confidentiality: any expression of self-harm or harm to others by any member, suspected child abuse or neglect, suspected elder abuse or neglect, suspected abuse of a disabled person
- All members have equal opportunity to speak and therefore members are expected to share the group’s time, take turns, thereby allowing other to speak and voice their opinions
- Do not multi-task, please be available to engage in the conversation/discussion for the time of the network
- Address each other with name

After the ground norms have been repeated, **please review any questions/comments from the last session that were not answered or were raised in the chat related to the recorded session.**

## Attachment 5 Permission to Record

At the start of each session, please review with the participants if they agree for the session to be recorded. If they do not agree or if you find that recordings interrupt with the open discussion, please inform the participants that the talk by guest presenters will still be recorded and will be available for them to view later. Inform the participants that a chat option is available in the web portal where the recorded sessions are saved. Any comments/questions in the chat will be reviewed and attended to in the next live PSN session.

## Attachment 6 Attendance Sheet

Please add as many rows to the table below as needed.

| Service name | Attendees |
| --- | --- |
|  |  |
|  |  |

## Attachment 7 Sample template to record meeting minutes


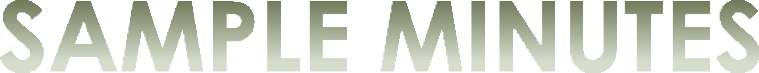

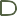


Aboriginal Informed Diabetes Training Program –

Peer Support Network

*Date* | Time, location *Zoom details:* Facilitator Name

| **Item** | **Discussion** | **Actions** |
| --- | --- | --- |
| 1 | Acknowledgement of Country |  |
| 2 | Welcome Attendees: Apologies: |  |
| 3 |  |  |
| 4 | Discussion |  |
| 5 | Announcement |  |
| 6 | Any other business |  |

## Attachment 8 Facilitator Reflection Form

The facilitator is required to complete this reflection form after each network session. It is recommended to complete the reflection form soon after the session.

#### Facilitator reflection form

Date of the session:

Date of completion of the reflection form:

- A detailed description of what was done in the peer support session?
- From the facilitator’s perspective, what went well in the session?
- From your perspective, what did not go well in the session?
  - What can be done to avoid that from happening in future?

## Attachment 9 Topics to be discussed in the network aligned with the AHW/P professional scope of practice

The topics given below are arranged in order of their appearance in the online modules. It is however recommended that the facilitator runs the first few sessions on their own and determine the sequence in which the topics are covered after discussion with the group.

| **Topics** |
| --- |
| Importance of registration with NDSS |
| Introduction to diabetes and how it develops including the role of pancreas, carbohydrates and glucose.  Types of diabetes (type 1, type 2, and gestational diabetes), risk factors of type 2 and gestational diabetes |
| Common symptoms of diabetes |
| Screening and diagnostic procedures |
| **Healthy living** |
| Healthy eating and healthy food choices (including carbohydrates)  Alcohol and smoking |
| Physical activity |
| **Glucose monitoring** |
| -Three methods of glucose monitoring  -Benefits of self-blood glucose monitoring, correct technique of self-blood glucose monitoring Blood glucose target levels  Benefits of a blood glucose action plan |
| Symptoms of hypoglycaemia, its management and prevention Symptoms of hyperglycaemia, its management and prevention  Benefits of a hypo and hyper/sickday action plan. |
| **Medicines and Insulin**  Promote quality of use of medicines  Storage and handling recommendations for insulin, none insulin injectables and oral medications.  Know the support person in the diabetes health care team for further discussion on medications (referral guide)  Benefits of a medication management action plan |
| **Diabetes related complications**  Know the types of diabetes related complications  Know how to prevent and identify diabetes related complications |

| List steps a client can take to reduce the risk of complications  It is possible to reduce the risk of developing diabetes related complications |
| --- |
| **Support for self-management**  Assist people with diabetes to identify the aims of diabetes management Main approaches to self-managing diabetes  **Impact of diabetes on a person’s emotional wellbeing ** can be a topic on its own**  Identify who in the diabetes health care team can assist clients with various diabetes management needs (referral)  **Selfcare/wellbeing of AHW/P** (arrange an appropriate professional to run this session) |
| **Priority groups**  Women with pre-existing diabetes planning to get pregnant Women with pre-existing diabetes who are pregnant Women with gestational diabetes  Children and young people with diabetes  People with cognitive and other physical impairment Managing diabetes in old population |
| **Role of the multidisciplinary team**  Understanding the role of the multidisciplinary team in the holistic care of clients with diabetes is important. As the participants of this network are from different health services, the member of the team would differ.  First the session can focus on understanding the role of the team members and then participants from each service can do a self-directed activity where they identify and fill in details of the multidisciplinary team members available within their region. These professionals are the ones that the AHW/Ps can refer their clients to.   - Aboriginal Health Workers & Practitioners - RNs & ENs - Medical Practitioners – GPs, GP Registrars - Diabetes nurse educator - Dietitian - Podiatrist - Psychologist - Credentialled diabetes educator |

| - Clinical specialists – cardiologist, endocrinologist, gastroenterologist & hepatologist, infectious diseases specialist, nephrologist - Other providers – social workers, case workers, alcohol & other drug workers, narrative therapists, counsellors - Providers may be internal or external to health service |
| --- |

## Attachment 8 Activities/Strategies to cover the topics and Topic Template

Topics given in the topic list of this guide may be covered by various strategies. Following are some suggestions (and templates) in which a topic may be presented to or discussed with the group.

### Case study – template on how to present a case study

### Stories/lived experiences – template

1. Guest speakers – contacting guest speaker template
2. Direct reflections
3. Readings – reading list
4. Case study – template on how to present a case study

A case study is an analysis of a real-life situation with some complexity or problem. The presenter’s job is to describe the case to the rest of the group. If it is a patient health/social issue related presentation, you will need to anonymise the information.

#### Structure

While putting a structure to your talk, think of a client presenting. Use that presentation sequence and information gathering that one would do with a client to structure and present a case study to the group.

- 1. Outline the purpose of the case study – have an introduction
  2. Identify the problems/issues
  3. Select the major issues in the case
  4. Solutions taken or that you think could be done about the problem
  5. The group may brainstorm to come up with solutions- **group discussion**
  6. Conclusion – summaries main points from the case presentation and group discussion
  7. Recommendations –are there any solutions that the group think are best/superior
  8. Implementation – what, who, how

#### Information gathering

In order to present a case study the presenter will need to gather some information. The information may come from medical notes – Information from the medical notes about client/patient presentation - history, information on relevant laboratory reports, etc. The information may be around any social issues – identified by the client. Present as much information as you can.

1. Stories/lived experiences – template

- Stories can be told using oral and/or visual material
- Participant/presenter synthesize and integrate their understanding of diabetes with a personal, cultural, and contextual narrative.
- Describe a way diabetes has affected your life
- How does a participant hopes to apply the knowledge as a result of participating in the peer support network or the whole diabetes training program to bring changes into your personal/family/professional life
- Participant may choose to tell stories related to for example their struggle/success with tobacco cessation, importance of healthy lifestyle and their struggle/success, importance of regular health checks (with or without a diagnosis of diabetes), loss, grief, self-care.
- Story can be told from perspective of being a caregiver or family member of a person with diabetes and self-care as a care provider
- Self-care as a healthcare provider
- Participants can include cultural perspectives about diabetes, reflect on traditional values, language, traditions, ways of knowing, intergenerational knowledge transmission, and other cultural attributes

Reference

Melany Cueva, Regina Kuhnley, Laura J. Revels, Katie Cueva, Mark Dignan & Anne P. Lanier. Bridging storytelling traditions with digital technology. Int J. of Circumpolar Health. 2013; 72 (1).

### Guest speakers – contacting the guest speaker

When a guest speaker is approached, the facilitator needs to introduce them to the purpose of the PSN. Also explain that the session is for open, fluid discussion on the topic and that the participants are encouraged during the session to ask questions and actively participate in the discussion.

It would help if the facilitator could give an estimate of time that the presenter will have if they are coming with a formal presentation.

Please contact the research team to get support in identifying guest speakers.

### Direct reflections template

Date:

Describe an activity that relates to your management of a client with diabetes, then reflect on your learning and professional growth. Explain what aspects of these experiences were most useful in your practice.

#### Activity:

#### Reflection:

- What was your thinking?
- What were your feelings?
- What sense can you make of the situation?
- What else could you have done?
- If a similar situation arises in future, would you act/behave/manage it in the same way?
- If a similar situation occurs in future, what would you do differently?

## Attachment 9 Tool kit Appendix

- 1. Evaluation survey (anonymous survey for the participants to complete)
  2. Research team’s responsibilities

### Evaluation survey (anonymous survey for the participants to complete at the end of each meeting)

The answers to the following questions will be used to modify the peer support network. Your opinions are important and will help in the improvement of the network so that it suits the needs of the AHW/Ps.

- 1. I enjoyed the session.

strongly disagree☐ Disagree☐ neither agree or disagree☐ agree☐ strongly agree☐

- 1. All participants were given a chance to have their say.

strongly disagree☐ Disagree☐ neither agree or disagree☐ agree☐ strongly agree☐

- 1. The participants of the network listened to one another.

strongly disagree☐ Disagree☐ neither agree or disagree☐ agree☐ strongly agree☐

- 1. What could be done better in the next meeting? Response: open ended
  2. The time of the session was suitable.

strongly disagree☐ Disagree☐ neither agree or disagree☐ agree☐ strongly agree☐

- 1. The duration of the session is appropriate.

strongly disagree☐ Disagree☐ neither agree or disagree☐ agree☐ strongly agree☐ Is there anything else that you would like to tell us?

### Research team’s responsibilities

- It is the research team’s responsibility to identify professional and reliable support for the facilitator and any other group members should the need arise.
- Help identify a list of potential guest speakers
- Meet with the facilitator to identify any challenges/barriers that can be dealt with within a reasonable timeframe to improve the network
