# Supplementary material for: Translation of culturally and contextually informed diabetes training for Aboriginal primary health care providers on Aboriginal client outcomes: Protocol of a cluster randomized crossover trial of effectiveness
Source: PLoS One. 2024 Jul 23;19(7):e0305472. doi: 10.1371/journal.pone.0305472 (PMC11265707; doi:10.1371/journal.pone.0305472)
Supplement: S5 File — (DOCX) [file pone.0305472.s005.docx]

Attachment 3


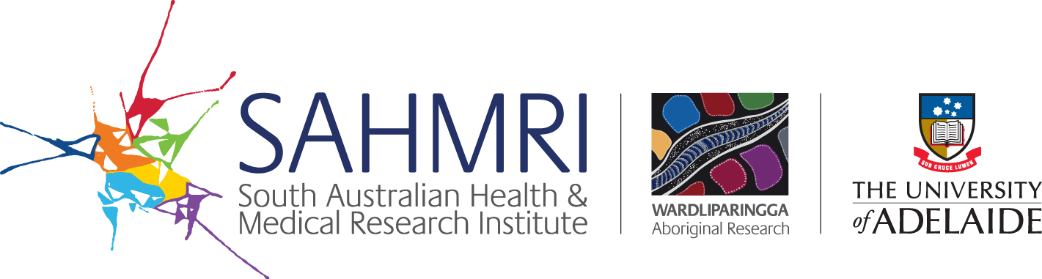


Aboriginal Informed Diabetes Training Program

ONSITE SUPPORT FACILITATOR

GUIDE

Contents

[Onsite Support – Guide 2](#_bookmark0)

[Examples of elective activities 2](#_bookmark1)

[Topics to be discussed in the network aligned with the AHW/P professional scope of practice 2](#_bookmark2)

[Evaluation 5](#_bookmark3)

1. [Facilitator reflection form— Onsite Support 5](#_bookmark4)
2. [Interview guide onsite support facilitator 5](#_bookmark5)
3. [Interview guide Aboriginal health worker/practitioner 5](#_bookmark6)

[Questions related to onsite support 6](#_bookmark7)

[Appendix 1 7](#_bookmark8)

[References 8](#_bookmark9)

# Onsite Support – Guide

The aim of the onsite support is to support the Aboriginal health worker/practitioners (AHW/Ps) with the translation of diabetes related knowledge and skills that they obtain via the other two components of the training program.

The onsite practice support component of the Aboriginal Informed Diabetes Training Program will be facilitated by a credentialed diabetes educator (CDE). A formal mentoring agreement outlining the expectations both from the mentor (facilitator) and mentee (AHW/P) will be completed prior to starting the onsite support component (Appendix 1).

## Examples of elective activities

The learning activities of the onsite support can be performed on any topics related to the diabetes educational content. A suggested topic list is included in this guide.

- Meeting to discuss technical information, in areas such as types of diabetes, healthy eating, physical activity, diabetes management.
- Prevention of diabetes related complications
- Foot check
- Identification of the role of multidisciplinary team and knowledge of referral pathway/person within the context of the health service.
- Hands-on support with performing health related tasks for clients with diabetes
- Discussing patient case studies
- Discussing about local Aboriginal history and culture
- Support around administrative tasks
- Planning health promotion events

## Topics to be discussed in the network aligned with the AHW/P professional scope of practice

The topics given below are arranged in order of their appearance in the online modules. It is however recommended that the facilitator runs the sessions in agreement with participants to meet their needs.

| **Topics** |
| --- |
| Importance of registration with NDSS |
| Introduction to diabetes and how it develops including the role of pancreas, carbohydrates and glucose.  Types of diabetes (type 1, type 2, and gestational diabetes), risk factors of type 2 and gestational diabetes |
| Common symptoms of diabetes |
| Screening and diagnostic procedures |
| **Healthy living** |

| Healthy eating and healthy food choices (including carbohydrates)  Alcohol and smoking |
| --- |
| Physical activity |
| **Glucose monitoring** |
| -Three methods of glucose monitoring  -Benefits of self-blood glucose monitoring, correct technique of self-blood glucose monitoring Blood glucose target levels  Benefits of a blood glucose action plan |
| Symptoms of hypoglycaemia, its management and prevention  Symptoms of hyperglycaemia, its management and prevention Benefits of a hypo and hyper/sickday action plan. |
| **Medicines and Insulin**  Promote quality of use of medicines  Storage and handling recommendations for insulin, none insulin injectables and oral medications.  Know the support person in the diabetes health care team for further discussion on medications (referral guide)  Benefits of a medication management action plan |
| **Diabetes related complications**  Know the types of diabetes related complications  Know how to prevent and identify diabetes related complications  List steps a client can take to reduce the risk of complications  It is possible to reduce the risk of developing diabetes related complications |
| **Support for self-management**  Assist people with diabetes to identify the aims of diabetes management Main approaches to self-managing diabetes  **Impact of diabetes on a person’s emotional wellbeing ** can be a topic on its own**  Identify who in the diabetes health care team can assist clients with various diabetes management needs (referral)  **Selfcare/wellbeing of AHW/P** (arrange an appropriate professional to run this session) |
| **Priority groups**  Women with pre-existing diabetes planning to get pregnant |

| Women with pre-existing diabetes who are pregnant Women with gestational diabetes  Children and young people with diabetes  People with cognitive and other physical impairment Managing diabetes in old population |
| --- |
| **Role of the multidisciplinary team**  Understanding the role of the multidisciplinary team in the holistic care of clients with diabetes is important. As the participants of this network are from different health services, the member of the team would differ.  First the session can focus on understanding the role of the team members and then participants from each service can do a self-directed activity where they identify and fill in details of the multidisciplinary team members available within their region. These professionals are the ones that the AHW/Ps can refer their clients to.   - Aboriginal Health Workers & Practitioners - RNs & ENs - Medical Practitioners – GPs, GP Registrars - Diabetes nurse educator - Dietitian - Podiatrist - Psychologist - Credentialled diabetes educator - Clinical specialists – cardiologist, endocrinologist, gastroenterologist & hepatologist, infectious diseases specialist, nephrologist - Other providers – social workers, case workers, alcohol & other drug workers, narrative therapists, counsellors - Providers may be internal or external to health service |

# Evaluation

Evaluation of this component will be performed by completion of a reflection form by the facilitator after each session (1. Facilitator reflection form – Onsite Support) and facilitator’s interview after completion of the study (2. Interview guide Onsite Support Facilitator).

AHW/Ps will also be interviewed for the process evaluation and to identify barriers and enablers to the sustainability of the onsite support after each session (3 Interview guide Aboriginal health worker/practitioner).

## Facilitator reflection form— Onsite Support

The facilitator is required to complete this impression form after each network session.

Date of the session:

Date of completion of the impression form:

- A detailed description of what was done in the onsite support session.
- From facilitator’s perspective, what went well in the session?
- From your perspective, what did not go well in the session?
  - What can be done to avoid that from happening in future?

## Interview guide onsite support facilitator

*Thank you for agreeing to be interviewed today. We are interested in talking to you to understand your experience as the onsite support facilitator. We also want to understand what you think worked well and what did not work well during the onsite support provision. The information you provide will help us improve the practice support provided to the AHW/Ps and make it sustainable for future. This interview will take approximately 30 minutes.*

- - What/how was your experience of facilitating the onsite support?
  - What are the key factors and process that support the AHW/Ps to actively participate in the onsite support?
  - What are the key factors and process that hinder the AHW/Ps to actively participate in the onsite support?
  - What are the key factors and process influencing the sustainability and ongoing operation of the onsite support for the study participants?
  - What are the key factors and processes that support study participants to apply the skills and knowledge they have gained from the Aboriginal Informed Diabetes Training Program within their local communities?
  - What are the key factors and processes influencing the sustainability and ongoing operation of the onsite support for AHW/Ps involved in the practice support component of the training?

## Interview guide Aboriginal health worker/practitioner

The AHW/Ps will be interviewed to capture their experience and feedback to the peer support network and the onsite support components of the Aboriginal Informed Diabetes Training Program.

*Thank you for agreeing to be interviewed today. We are interested in talking to you to understand your experience as the participant of the Aboriginal Informed Diabetes Training Program. We also want to understand what you think worked well and what did not work well during the training program. The information you provide will help us improve the diabetes training provided to the AHW/Ps and make it sustainable for future. This interview will take up to 1 hour.*

I have a list of questions that were put together to guide our discussion today, *but you can yarn with us in any way that feels right for you.*

- - There are three components of the Aboriginal Informed Diabetes Training Program, peer support network, onsite support, and the online module. Which components of the training program did you participate in?

Questions related to onsite support

- - What/how was your experience of participating in the onsite support?
  - What are the key factors and process that enabled you to actively participate in the onsite support?
  - What are the key factors and process that made it difficult to actively participate in the onsite support?
  - What are the key factors and process influencing the sustainability and ongoing operation of the onsite support for the study participants?

# Appendix 1

### Aboriginal Informed Diabetes Training Program Onsite Support

### Mentoring Agreement

This agreement is between:

Name of mentor (facilitator):

Name of mentee (AHW/P):

Position title:

Health Service:

Likely frequency of meetings

Proposed length of each meeting

### What 3 areas would you like to cover in the onsite support?

**Aim** of the onsite support is to assist AHW/Ps’ knowledge translation on the management and prevention of diabetes for their clients within the Aboriginal communities that they work in. Obtaining practice support by the CDE is a voluntary process of building a trusting, mutually beneficial learning relationship between professionals that supports the development of knowledge, skills, attitudes and behaviours in order to reach important career goals.

### Responsibilities of AHW/P

- - Identify your learning and professional needs
  - Self-assess your current competency in relation to a set of defined core competencies in the management of diabetes for AHW/Ps
  - Schedule uninterrupted time to meet with your mentor
  - Actively engage in the mentoring program through participation in individual/group mentoring sessions
  - Undertake professional development tasks within agreed timelines
  - Communicate with your mentor, listen and ask questions as required for the onsite support
  - Ensure that any action you take as a result of mentoring is ethical and appropriate for your organisation and community
  - Maintain confidentiality regarding personal information revealed during the mentoring process
  - Participate in process evaluation at the conclusion of the onsite support

### Responsibilities of Facilitator

- - Discuss career/professional goals with AHW/P
  - Develop mutually agreed goals for the onsite support with the AHW/Ps
  - Schedule uninterrupted time to meet with AHW/Ps
  - Work with AHW/Ps to plan strategies to achieve the agreed upon goals
  - Maintain confidentiality
  - Respect the cultural experience and knowledge of AHW/Ps
  - Encourage and maintain two-way communication with AHW/Ps
  - Maintain regular contact with research team
  - Participate in project evaluation at the conclusion of the onsite support

# References

S Thorpe, J Browne. Heart health action in Aboriginal communities: translating training into practice, Final evaluation report.
