# Supplementary material for: Translation of culturally and contextually informed diabetes training for Aboriginal primary health care providers on Aboriginal client outcomes: Protocol of a cluster randomized crossover trial of effectiveness
Source: PLoS One. 2024 Jul 23;19(7):e0305472. doi: 10.1371/journal.pone.0305472 (PMC11265707; doi:10.1371/journal.pone.0305472)
Supplement: S6 File — (DOCX) [file pone.0305472.s006.docx]

# Overview

An outline of data collection methods is provided for demographic, primary and secondary outcomes and potential confounding variables.

# Data collection timepoints

For the entire study there are seven points in time at which data will be collected. These are labelled T0 (baseline), T1 – T6 in Figure 4 and are referred to in this document.


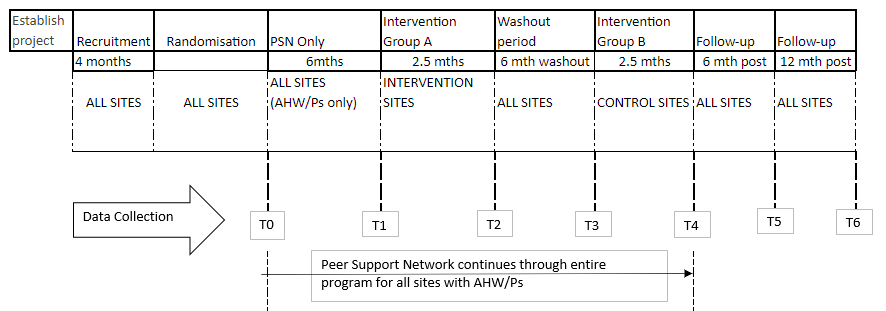


Figure 1: Data collection timepoints

Contents

[Overview 1](#_Toc149731511)

[Data collection timepoints 1](#_Toc149731512)

[Participant demographics 1](#_Toc149731513)

[Primary outcome measure – Practice & Skills 4](#_Toc149731514)

[Primary outcome measure – Skill (diabetes foot check) 7](#_Toc149731515)

[Secondary measure: Peer Support Network satisfaction survey 10](#_Toc149731516)

[Secondary measure: Enablers and barriers to participation, effectiveness and sustainability of the training program 11](#_Toc149731517)

[Secondary measure: Quality and outcomes of diabetes care 12](#_Toc149731518)

[Potential confounding variables 15](#_Toc149731519)

# Participant demographics

| **Demographics** | | |
| --- | --- | --- |
| **Data Source** | All participants: Aboriginal Health Workers, Aboriginal Health Practitioners, Multi-disciplinary staff | |
| **Data collection method** | RedCap Survey | |
| **Collection timeframe** | 14 days | |
| **Time points for collection** | T0, T5, T6 | |
| **Informed by a validated tool** | Research team/Investigator defined | |
| **Level of collection** | Individual | |
| **Rationale** | To identify participant characteristics relevant to training and provision of diabetes care | |
| **Variables** | | |
| Which Aboriginal health service/s do you currently work at? | | Please select all that apply  [names of health service, drop down box] |
| Are you: | | 1, an employee of this health service?  2, a visiting service provider? |
| What is your current role? | | 1, Aboriginal Health Worker  2, Aboriginal Health Practitioner  3, Enrolled Nurse  4, Diabetes Educator  5, Nurse  6, Allied Health Professional  7, Medical Practitioner  9, Other with free text box |
| How long have you worked in your current role? | | 1, <1 year  2, 1-5 years  3, 5-10 years  4, >10 years  5, Prefer not to say |
| How long have you worked with Aboriginal and Torres Strait Islander clients? | | 1, <1 year  2, 1-5 years  3, 5-10 years  4, >10 years |
| What age group are you in? | | 1, 18-24 years  2, 25-34 years  3, 35-39 years  4, 40-44 years  5, 45-49 years  6, 50-54 years  7, 55-59 years  8, 60-64 years  9, 65+ years  10, Prefer not to say |
| Gender Identity | | 1, Male  2, Female  3, Prefer not to say  4, Other |
| Are you of Aboriginal or Torres Strait Islander origin? | | 0, No  1, Yes, Aboriginal  2, Yes, Torres Strait Islander  3, Yes, both Aboriginal and Torres Strait Islander  4, Prefer not to say |
| What is your highest level of education completed? | | 0, None  1, High school  2, Certificate/Diploma  3, University  4, Other (with free text box) |
| Have you completed the Aboriginal and Torres Strait Islander health - diabetes e-Learning modules available on the NDSS (National Diabetes Services Scheme) website? | | 1. Yes 2. No |
| If so, please attach completion certificate for the NDSS e-Learning modules | |  |

# Primary outcome measure – Practice & Skills

| **Primary Outcome Measure – Practice & Skills** | | |
| --- | --- | --- |
| **Data Source** | All participants: Aboriginal Health Workers, Aboriginal Health Practitioners, Multi-disciplinary staff | |
| **Data collection method** | RedCap Survey | |
| **Collection timeframe** | 14 days | |
| **Time points for collection** | T0 – T6 | |
| **Informed by a validated tool** | Research Team/Investigator informed | |
| **Level of collection** | Individual | |
| **Rationale** | To evaluate the effectiveness of the training program on participant **practice and skill** in managing diabetes | |
| **Case Studies** | | |
| Question | | Answers |
| 1. You are seeing a 60-year-old person who has come to review their lab results today. At the last visit about a month ago, this patient presented for a medical exam and told you that they had not seen a doctor for last 5 years. There is also a complaint of getting up to the toilet much more often during the night, feeling thirsty. The results of the investigations have returned and fasting blood glucose level is 8mmol/L, based on which a diagnosis of diabetes is made. You obtain a weight (90kg), height (172cm) and waist circumference (104cm). Which of the following is the most appropriate approach to initiate a discussion about these findings? | | - 1. Advise that the person needs to lose weight and refer to an endocrinologist for assessment and management   2. Tell the patient that their diabetes is because of obesity and prescribe metformin and a GLP1 agonist   3. Inform the person that they have type 2 diabetes and ask if they are willing to talk about the sorts of foods he is eating and what he drinks   4. Tell the person that their diet is a problem and refer to a dietitian   Correct answer is c |
| 1. One of your patients has been coming to see you for a while and seems to have made little progress with weight loss and therefore their type 2 diabetes is not well controlled. You wonder why and decide to ask some questions and encourage better engagement. Which one of the following will be best to start the discussion? | | - 1. We are often surrounded by various unhealthy food options how do you manage to resist temptation.   2. I know that you have a lot of friends and a very active social life. Do you find it a problem not to overindulge?   3. You have a very busy life with lots of pressures. What strategies could you use to be more disciplined about the snacking?   4. What are the situations that you find create challenges or difficulties in staying on track?   Correct answer is d |
| 1. A patient, whose patient record shows has type 2 diabetes, attends the clinic for a routine visit. Your initial impression is that the patient is severely obese. Which of the following is the most appropriate approach to initiate a discussion about obesity? | | - 1. Casually draw attention to the problem with reference to the need for a very large cuff to measure blood -pressure.   2. Break any tension about weight being an issue with a light-hearted comment before directly introducing the subject.   3. Complete your assessment and indicate you would like to discuss the various factors relating to living well with type 2 diabetes. Ask if the patient feels comfortable discussing their weight with you.   4. Make it clear at the outset that excess weight is related to diabetes and it is something that has got to be dealt with   Correct answer is c |
| 1. A female AHW/P sees a male patient who has agreed to a general triage prior to seeing a doctor. He seems irritable and moody and seems reluctant to talk further. You suspect that he is bothered by some specific issues. Which of the following is the most appropriate action? | | - 1. Ask directly what other issues he have.   2. Ignore and do not try to interfere in personal business.   3. Ask the patient if they would prefer to see a male AHW/P for a confidential discussion and make referral.   4. Hand him some information about depression, sexual function and diabetes.   Correct answer is c |
| 1. A male AHEW/P sees a female patient who has agreed to a general triage before seeing a doctor. The patient seemed shy, uncomfortable, and not engaging with the practitioner. You suspect that there are some underlying issues bothering the patient. What would be the most appropriate steps to encourage patient engagement? | | - 1. Ask the patient if she has any cultural believes that need to be considered for her care   2. Ask directly what issues she has   3. Ask the patient if she would feel more comfortable to have an appointment with a female health professional   4. Hand some information about urinary tract infections, sexual function and diabetes.   Correct answer is c |
| 1. You are seeing a 28-year-old person in clinic today, who has been prescribed insulin to manage type 1 diabetes. Which of the following is the most important information to ensure they know about the use of insulin? | | - 1. The risk of low blood sugars and strategy to manage this   2. Insulin is best injected in the same place each time   3. Insulin is completely safe to use and there are not precautions needed   4. When too sick to eat or vomiting it is okay to miss an insulin dose to avoid hypoglycaemia.   Correct answer is a |
| 1. The 28-year-old that you saw in clinic, using insulin, is going out camping for the weekend. You noticed that the temperature is going to be above 30 degrees Celsius, and the patient will have to carry insulin for use. What would you recommend? | | - 1. Insulin is stable and can be kept safely in the car.   2. Keep insulin in a cool insulated bag between 4 and 28 degrees Celsius.   3. It is unsafe for people with type 1 diabetes to go camping.   4. Replace the insulin with oral medications while camping.   Correct answer is b |
| 1. You are seeing a 50-year-old person in clinic today, you found that they have been prescribed long acting insulin glargine 100 units/mL, 16 units at bedtime to manage their type 2 diabetes. Which of the following is the most important information that this person needs to know about the use of insulin? | | - 1. Insulin use has put this person at risk of developing hypoglycaemia and that they should seek to have a hypoglycaemia action which will include what fast-acting carbohydrates to have handy at all times and what follow up action is needed.   2. Insulin use does not mean that diabetes has progressed to a level where their body cannot cope   3. Insulin use does not have any side effects   4. It is possible to have an extra meal by increasing insulin dose   Correct answer is a |
| 1. You are seeing a 65 year old person who lives alone and is struggling with multiple medications, prescribed for diabetes and other health problems, which have to be taken at different times. How can you best assist them with their medication intake? | | 1. Arrange for a Webster pack and Home Medicines Review 2. Discuss the risks and benefits of each medication and provide written advice. 3. Write a letter to their GP explaining the problem 4. It is best not to interfere as the GP takes care of the medications.   Correct answer is a |

# Primary outcome measure – Skill (diabetes foot check)

| **Primary Outcome Measure – Skill (diabetes foot check)** | | |
| --- | --- | --- |
| **Data Source** | All participants: Aboriginal Health Workers, Aboriginal Health Practitioners, Multi-disciplinary staff | |
| **Data collection method** | RedCap Survey | |
| **Collection timeframe** | 14days | |
| **Time points for collection** | T0 – T6 | |
| **Informed by a validated tool** | Adopted from the Foot Forward Project | |
| **Level of collection** | Individual | |
| **Rationale** | To evaluate the effectiveness of the training program on participant **skills** in performing a foot check | |
| **Case Study**  Jodie is a 59-year-old Aboriginal woman who is a regular client of the local Aboriginal health service. Jodie was diagnosed with diabetes 18 months ago. So far, she had been able manage her blood glucose levels by adjusting her diet and exercising four times a week.  Jodie has attended today for a regular six moth review as part of her annual cycle of care for diabetes. When you call Jodie in, she is pleased to tell you that she has not noticed any signs or symptoms of diabetes and is generally feeling well. She is enjoying exercising regularly and is planning to keep it up. Her observations are all within the normal range.  All questions in the below section are related to the case given above. | | |
| Variable | | Multiple Choice Answers |
| You explain to Jodie that you would like to conduct a foot check. Which of the following do you need to explain in order to help Jodie understand why a foot check is important and what will be done with the results: | | 1. That a foot check will help to understand how diabetes had affected her feet 2. That the foot check will help the healthcare team to provide appropriate care 3. That if there are any concerns, she will be referred to an appropriate clinician, such as the GP or podiatrist 4. All of these   Answer d) |
| Jodie is happy to have had the process explained and readily agrees to having a foot check. Your health service has 10-gram (5.07 Semmes-Weinstein) monofilaments and you decide to conduct a monofilament test.  When doing a 10-gram monofilament test, what are you checking Jodie for? | | 1. Change in colour of the skin 2. Loss of protective sensation 3. Skin temperature – a change in skin temperature – cold or hot 4. Swelling or oedema   Answer b) |
| Checking for loss of protective sensation (LOPS) is important for people with diabetes because: | | 1. Diabetes-related peripheral neuropathy can cause LOPS and interfere with a person’s ability to feel pain, so that they may not be aware of a wound developing or becoming infected. 2. If a person cannot fell when they have an injury to their foot or a foot ulcer, they are at greater risk of the injury or ulcer becoming infected. Most amputations in people with diabetes are because a foot ulcer has become severely infected. 3. If Jodie has LOPS, the healthcare team will work with Jodie to schedule more regular checks so that any foot problems, such as callus or early signs of foot ulcers can be identified early and properly treated to prevent complications, such as amputations. 4. All of these.   Answer a) |
| 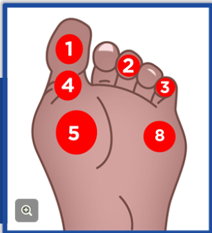  When doing a 10-gram monofilament test, which parts of Jodie’s feet are you going to check? | | 1. 1, 5, 8 2. 1, 2, 3 3. 1, 2, 4 4. 4, 5, 8   Answer a) |
| After explaining to Jodie how the test works and demonstrating the feeling of the monofilament, you proceed to conduct the test. You found that Jodie has a loss of feeling on point 1 and 5 in her right foot.  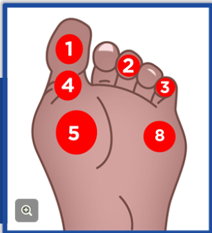  What is the next thing you should do? | | 1. Tell Jodie what the findings are and explain that you are going to refer her to the podiatrist or general practitioner. 2. After considering your options you decide not to worry Jodie by telling her, however, you tell the registered nurse who can manage it from here. 3. You think you have probably done the test incorrectly as Jodie seems very well and is managing her diabetes well. You decide to wait until her next 6 monthly review to check again. 4. You tell Jodie that this is a very serious finding and she is likely to require an amputation unless action is immediately taken.   Answer a) |
| You proceed with the rest of the foot check. You notice this on Jodie’s big tow. What is it likely to be?  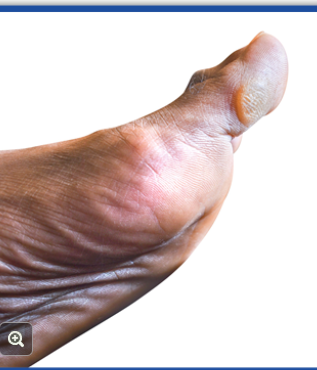 | | 1. A callus 2. A foot ulcer 3. Fungus 4. Nothing   Answer a) |
| After documenting the callus and completing the physical foot check, what is the next step? | | 1. Ask Jodie to put her shoes back on and proceed to make appointments for follow-up 2. Check Jodie’s footwear 3. Ask Jodie to return for her usual check in 6 month’s time 4. Explain to Jodie that she is at very high risk of developing foot ulcers and that she needs to see a vascular surgeon as soon as possible   Answer b) |
| You now go on to check Jodie’s footwear.  What it the next thing you do? | | 1. Complete the consultation and ask Jodie to make her appointment with the GP or podiatrist 2. Identify Jodie’s self-care capacity 3. Tell Jodie that trying new footwear is likely to solve any foot problems that she may have and that she will be reminded of her next appointment in 6 months’ time 4. Ask Jodie to return tomorrow as you have run out of time to complete the foot check   Answer b) |
| You have assessed Jodie’s self-care capacity. You have demonstrated to Jodie how to clean, dry and moisturise her feet daily. You have also recommended that she use a handheld mirror to see under her feet properly when se is examining her feet daily.  You contacted reception and secured an appointment for Jodie with her general practitioner for tomorrow.  Before ending the consultation, you decide to give Jodie three key messages about caring for her feet. What are the three most important things you could advise Jodie? | | 1. To be sure to attend for her appointment with the general practitioner tomorrow, and if she cannot make it to the appointment to call and make another one as her LOPS test results and callus need to reviewed by her doctor 2. To follow through on her purchasing new shoes that will be more appropriate for her and that will fit her lifestyle 3. To continue her daily foot care and if she finds any changes in her feet to contact her health service 4. All of these   Answer d) |

# Secondary measure: Peer Support Network satisfaction survey

| **Secondary outcome measures –Peers Support Network Satisfaction Evaluation Survey.** | | |
| --- | --- | --- |
| **Data Source** | Peer Support Network Participants (Aboriginal Health Workers, Aboriginal Health Practitioners) | |
| **Data collection method** | RedCap Survey | |
| **Collection timeframe** | 14 days | |
| **Time points for collection** | Monthly after each Peer Support Network Meeting | |
| **Informed by a validated tool** | Informed by a scoping review of the literature on communities of practice | |
| **Level of collection** | Individual | |
| **Rationale** | To continually improve the PSN by responding to positive suggestions and addressing barriers and evaluate the PSN. | |
|  | | |
| **VARIABLES** | | **ANSWERS** |
| I enjoyed the PSN session* | | 1 = Strongly agree; 2 = Agree; 3 = Neither agree nor disagree; 4 = Disagree; 5 = Strongly disagree |
| All participants in the PSN session were given a chance to have their say | | 1 = Strongly agree; 2 = Agree; 3 = Neither agree nor disagree; 4 = Disagree; 5 = Strongly disagree |
| Participants in the PSN session listened to one another | | 1 = Strongly agree; 2 = Agree; 3 = Neither agree nor disagree; 4 = Disagree; 5 = Strongly disagree |
| What do you think could be done better in the next PSN session? | | (text box to write open ended response) |
| The scheduled time of the PSN session was suitable for me to attend | | 1 = Strongly agree; 2 = Agree; 3 = Neither agree nor disagree; 4 = Disagree; 5 = Strongly disagree |
| The duration of the PSN session was appropriate | | 1 = Strongly agree; 2 = Agree; 3 = Neither agree nor disagree; 4 = Disagree; 5 = Strongly disagree |
| Is there anything else that you would like to add about your experience of the PSN session today? | | (text box to write open ended response) |
| I was able to join PSN without any technology related interruptions.* | | 1 = Strongly agree; 2 = Agree; 3 = Neither agree nor disagree; 4 = Disagree; 5 = Strongly disagree |
| The topics discussed in the PSN session would be useful in my work * | | 1 = Strongly agree; 2 = Agree; 3 = Neither agree nor disagree; 4 = Disagree; 5 = Strongly disagree |

*Also potential confounding variables

# Secondary measure: Enablers and barriers to participation, effectiveness and sustainability of the training program

| **Secondary outcome measures – enablers and barriers to participation, effectiveness and sustainability of the training program** | |
| --- | --- |
| **Data Source** | A cross section of participants from each discipline; 3 to 5 with a total of up to 15 participants. |
| **Data collection method** | Semi-structured interview |
| **Collection timeframe** | 2 months |
| **Time points for collection** | Once during the study at T2 with Group A and T4 with Group B |
| **Informed by a validated tool** | Informed by a scoping review of the literature on chronic disease training programs for Community Health Practitioners |
| **Level of collection** | Individual |
| **Rationale** | To identify enablers and barriers to be able to inform improvements in the training program and implement strategies for sustainability of the training program. |
| **VARIABLES** | |
| What/how was your experience of participating in the PSN?* | |
| What are the key factors and processes that supported you to actively participate in the PSN?* | |
| What are the key factors and processes that prevented or made it difficult for you to actively participate in the PSN?* | |
| What are the key factors and process influencing the sustainability and ongoing operation of the PSN for Aboriginal Health Workers and Practitioners?* | |
| What/how was your experience of participating in the onsite support? | |
| What are the key factors and process that enabled you to actively participate in the onsite support? | |
| What are the key factors and process that made it difficult to actively participate in the onsite support? | |
| Can you describe your experience of being involved in the diabetes training program? | |
| From your perspective what was good about the program and what could be done to improve the program? | |
| What impact has the training program had on your ability to provide diabetes care to clients? | |
| Would you recommend the diabetes training program to Health Workers and Practitioners? | |
| On a scale of 1 to 7, how much do you think your knowledge of diabetes care has changed due to participating in the diabetes training program?  1 (not at all)  2 (slightly)  3 (somewhat)  4 (a moderate amount)  5 (a great deal)  6 (extremely)  7 (exceedingly) | |

# Secondary measure: Quality and outcomes of diabetes care

|  |  | |
| --- | --- | --- |
| **Data Source** | Electronic patient information system | |
| **Data collection method** | Automated data extraction using a data extraction algorithm, retrospective data collection at three timepoints during the study T | |
| **Collection timeframe** | Jan 2018 – 12 months post intervention | |
| **Time points for collection** | T1, T4, T6 | |
| **Informed by a validated tool** | Informed by best practice clinical care guidelines – assessed against RACGP evidence-based care guidelines | |
| **Level of collection** | Health Service & Individual patient with type 2 diabetes | |
| **Rationale** | To evaluate the impact of the training program on the quality of diabetes care provided and patient outcomes. | |
| **Name/Description of data** | Electronic Medical Records | Variable |
| **Data Custodian** | SA Health Primary Health Care Sites - Watto Purrunna (Muna Paiendi, Wonggangga Turtpandi), Aboriginal Family Clinic (Noarlunga, Clovelly Park), Yorke & Northern LHN (Point Pearce Port Pirie), Riverland Mallee Coorong LHN. |  |
| **Agency Type** | State government Aboriginal primary care service |  |
| **Data Collection Format** | De-identifiable |  |
| *Variable* | *Justification* |  |
| *Health service level* | To provide a basic description / service context for each service. |  |
| *Regular client* | Defined as 3 or more visits in the previous 2 years. This will become the denominator for the proportion of clients with diabetes within a service. | Whole number and date at which identified |
| *Number of regular and transient clients on a care plan* | The number of current/regular and the number of transient Aboriginal clients, registered with the following conditions, including the number in each category and disease group with a current disease-specific care plan.   - Cardiovascular Disease - Renal Disease - Mental Health - Diabetes | Whole number and date at which identified |
| *Number of transient clients* | The number of clients who are defined as a transient client receiving care at the health service. Transience defined as < 2 visits in 12 months. | Whole number and date at which identified |
| *Indigenous status* | Proportion of Aboriginal clients using the service of total client population | Proportion calculated by the services and date at which identified |
| *Episodes of care* | The number of episodes of care received by Aboriginal clients in the previous 12 months. An episode of care is defined as a presentation to the health service where a service was provided. | Whole number and date at which identified |
| *Client level* | Eligibility criteria: People with diabetes who have attended the participating health service and who fulfil the following criteria at the time of record review meet the following criteria:  aged18 years and over;  identify as Aboriginal;  have a confirmed diagnosis of T2DM and/or a HbA1c ≥6.5 mmol/L;  have attended the health service at least twice in the preceding 12-month period;  Those who meet the eligibility criteria become the study population in which the below variables are extracted. | Date at which study population is defined |
| *Client ID* | Service unique record ID created and provided by the health service so that no identifying data is provided to the study team |  |
| *Month, Year of Birth* | To estimate age |  |
| *Sex* | To examine gender differences |  |
| *Ethnicity* | To identify Aboriginal status |  |
| *GP Management Plan* | To calculate number of clients who have a GPMP | Date of last review |
| *Team Care Arrangements* | To calculate number of clients who have a TCA | Date of last review |
| *GP Management Plan Review* | To identify proportion who have had a GPMP review within the recommended timeframe according to evidence-based care guidelines | Date of last review |
| *Diabetic retinal check performed* | To identify proportion who have had a retinal check review within the recommended timeframe according to evidence-based care guidelines | Date of last review |
| *Diabetic foot check performed* | To identify proportion who have had a foot review within the recommended timeframe according to evidence-based care guidelines | Date of last review |
| *Absolute CV risk assessment* | To identify proportion who have had a CV risk assessment within the recommended timeframe according to evidence-based care guidelines | Date of last review |
| *BMI* | Identify proportion within recommended range | Most recent result and date |
| *Waist circumference* | Identify proportion within recommended range | Most recent result and date |
| *Smoking status* | Understand proportion of clients with behavioural risk factors | Most recent result and date |
| *Physical activity* | Understand proportion of clients with behavioural risk factors | Most recent result and date |
| *Influenza vaccination up to date* | Vaccination status | Date of last vaccination |
| *Pneumococcal vaccination up to date* | Vaccination status | Date of last vaccination |
| *Covid-19 vaccination* | Vaccination status | Date of last vaccination |
| *Clinical measurements:*  *Cholesterol (mmol/L)*  *LDL (mmol/L)*  *HDL (mmol/L)*  *Triglycerides (mmol/L)*  *Creatinine (μmol/L)*  *eGFR (ml/min/1.73m²)*  *uACR (mg/mmol)*  *Random BGL (mmol/L) - non-fasting, blood glucose test performed at the health service*  *HbA1c (mmol/mol)*  *Systolic BPM (mm/Hg)*  *Diastolic BPM (mm/Hg)* | Identify proportion within recommended range | Most recent result and date |
| *Number of failed follow up attempts in past 12 months* | Service utilisation |  |
| *Related conditions of clients with diabetes:*  *Coronary heart disease*  *Stroke or TIA*  *Peripheral artery disease*  *Congestive heart failure*  *Chronic kidney disease*  *Depression*  *Hypertension*  *Dyslipidaema*  *Cardiac dysrhythmia* | Understand burden of chronic disease among clients. | Y/N |
| *Referrals to diabetes educator, diabetes specialist, optometrist, podiatrist, dietician, dentist, psychologist, social worker, exercise physiologist, mental health screening* | Understand quality of care referral | Most recent date of referral |

# Potential confounding variables

| **Confounding variables** | | | | | |
| --- | --- | --- | --- | --- | --- |
| **Data Source** | | Varied | | | |
| **Data collection method** | | RedCap Survey | | | |
| **Collection timeframe** | | Varied | | | |
| **Time points for collection** | | Varied | | | |
| **Informed by a validated tool** | | Research team/Investigator defined using Directed Acyclic Graphs | | | |
| **Level of collection** | | Individual | | | |
| **Rationale** | | To control for bias in statistical models | | | |
|  | | | | |  |
| Variable | Data source (collected from) | | Frequency | Timepoint for collection | Notes |
| Have you completed the diabetes e-learning modules available from the NDSS website?  Yes/No/Other (open text option) | AHW/P  Multi-disciplinary team members | | Once | **B**aseline | This question is included in the demographic variables |
| Are you able to provide a completion certificate if asked to do so?  Yes/No/Other (open text option) | AHW/P  Multi-disciplinary team members | | Once | Baseline | This question is included in the demographic variables |
| I did not have any trouble accessing a computer to access the e-learning modules whenever I needed to.  Strongly disagree  Disagree  Neither agree nor disagree  Agree  Strongly Agree | AHW/P  Multi-disciplinary team members | | Once | After each intervention period for both groups |  |
| I was able to easily access the e-Learning modules (e.g. login details, internet speed).  Strongly disagree  Disagree  Neither agree nor disagree  Agree  Strongly Agree | AHW/P  Multi-disciplinary team members | | Once | After the intervention period for both groups |  |
| I was able to join PSN without any technology related interruptions  Strongly disagree  Disagree  Neither agree nor disagree  Agree  Strongly Agree | AHW/P | | Once a month (approx. 17 times) | After each PSN session | This question is already included in the post-PSN questions |
| I enjoyed the PSN session  Strongly disagree  Disagree  Neither agree nor disagree  Agree  Strongly Agree | AHW/P | | Once a month (approx. 17 times) | After each PSN session | This question is already included in the post-PSN questions |
| The topics discussed in the PSN session would be useful in my work  Strongly disagree  Disagree  Neither agree nor disagree  Agree  Strongly Agree | AHW/P | | Once a month (approx. 17 times) | After each PSN session | This question is already included in the post-PSN questions |
| Overall, I was satisfied with the content of the Diabetes e-Learning Modules.  Strongly disagree  Disagree  Neither agree nor disagree  Agree  Strongly Agree | AHW/P  Multi-disciplinary team members | | **Once** | After the intervention period for both groups |  |
| The topics discussed in Onsite Support session are likely to help me manage clients with diabetes.  Strongly disagree  Disagree  Neither agree nor disagree  Agree  Strongly Agree | AHW/P  Multi-disciplinary team members | | **Once** | After the intervention period for both groups |  |
