# Supplementary material for: Translation of culturally and contextually informed diabetes training for Aboriginal primary health care providers on Aboriginal client outcomes: Protocol of a cluster randomized crossover trial of effectiveness
Source: PLoS One. 2024 Jul 23;19(7):e0305472. doi: 10.1371/journal.pone.0305472 (PMC11265707; doi:10.1371/journal.pone.0305472)
Supplement: S7 File — (PDF) [file pone.0305472.s007.pdf]

RESEARCH PROTOCOL

Full Title: Translation of culturally and contextually informed diabetes training for Aboriginal primary health care providers on Aboriginal client outcomes: a cluster randomised trial of effectiveness

Short Title: The Aboriginal Diabetes Workforce Study

ABBREVIATIONS

Abbreviation	Explanation
AHW/P	Aboriginal Health Worker/Practitioner
ACIC	Assessment of Chronic Care Scale
CDE	Credentialled Diabetes Educator
DAS	Diabetes Attitude Scale
DAG	Directed Acyclic Graph
DCS	Diabetes Confidence Survey
DNE	Diabetes Nurse Educator
GLMMs	Generalized linear mixed effects models
NDSS	National Diabetes Services Scheme
OS	Onsite Support – one of three elements of the training program
PSN	Peer Support Network – one of three elements of the training program
RACGP	Royal College of Australian General Practitioners
SAHMRI	South Australian Health & Medical Research Institute
URID	Unique Record ID

CONTENTS

2. INTRODUCTION ... 5
3. BACKGROUND ... 5
4. AIM & OBJECTIVES ... 7
5. HYPOTHESIS ... 8
6. STUDY DESIGN ... 9
7. STUDY SETTING ... 10
8. STUDY POPULATION AND SETTING ... 10
9. ELIGIBILTY CRITERIA ... 11
10. STUDY OUTCOMES ... 12
11. STUDY PROCEDURES ... 12
12. DATA ANALYSIS ... 29
13. DATA HANDLING AND RECORD KEEPING ... 31
14. PUBLICATION & INTELLECTUAL PROPERTY ... 32
15. ETHICAL CONSIDERATIONS ... 33
16. OUTCOMES AND SIGNIFICANCE ... 36
17. REFERENCES ... 37

TABLES

Table 1: Study sites ... 10
Table 2: Time commitment ... 20
Table 3: Summary of data collection methods ... 22

FIGURES

Figure 1: Diabetes workforce training program ... 7
Figure 2: Study design ... 9
Figure 3: Study procedures flow diagram ... 17
Figure 4: Data collection timepoints ... 22

ATTACHMENTS

Attachment 1: Recruitment Email for CEO – Training Program

Attachment 2: Recruitment Email & Follow-up - Interviews

Attachment 3: Recruitment Email - System Assessment

Attachment 4: Participant Information Sheet – Training Program

Attachment 5: Participant Consent Form – Training Program

Attachment 6: Participant Information Sheet – Interviews Barriers and Enablers

Attachment 7: Participant Consent Form – Interviews Barriers and Enablers

Attachment 8: Participant Information Sheet – System Assessment

Attachment 9: Participant Consent Form – System Assessment

Attachment 10: Collaborative Agreement Template

Attachment 11: Communication during the intervention

Attachment 12: Example Friday update email to participants

Attachment 13: Participant Withdrawal Form

Attachment 14: Data Collection Methods and Variables

Attachment 15: Distress Protocol Peer Support Network

Attachment 16: Distress Protocol Onsite Support

Attachment 17: Distress Protocol Interviews

Attachment 18: Distress Protocol System Assessment

1. INTRODUCTION

The Aboriginal Diabetes Workforce Study (the study) involves co-designing, implementing and evaluating an Aboriginal Diabetes Workforce Training Program (training program) with health professionals employed by Aboriginal Primary Health Care Services in South Australia. This study is a direct response to several workforce strategies within the SA Aboriginal Diabetes Strategy 2017-2021 and that remain a priority based on recent discussions with Aboriginal primary health care services workforce.

During 2021, seven Aboriginal Primary Health Care Services across South Australia co-designed the training program with the research team, including the study investigators who were highly engaged and industry partners. Industry partners, Diabetes Queensland and Diabetes Australia and funding body by the NDSS, led the development of the e-learning modules. The training program consists of three elements: 1) Statewide Peer Support Network for Aboriginal Health Workers and Practitioners (AHW/Ps), 2) self-paced e-Learning modules, 3) Onsite Practice Support.

A cluster randomised control design at the service level will be used to evaluate the effectiveness of the training program on knowledge, confidence, attitude, practice and skill related to diabetes management, of health care providers. Health care providers include Aboriginal Health Workers, Aboriginal Health Practitioners and multidisciplinary health care providers working in Aboriginal primary health care services across South Australia. Implementing the training program has the potential to embed a minimum standard of diabetes training across the state and professionally develop and support the Aboriginal Health Worker and Practitioner workforce, vital in managing diabetes in the Aboriginal community.

2. BACKGROUND

Noncommunicable diseases account for 70% of deaths globally, with 4% of those deaths attributable to diabetes.[1] Well controlled diabetes is associated with increased longevity and prevention or slowing the progression of diabetes-related complications. Large randomised controlled trials have found that a reduction in HbA1c by 1% is associated with a 10% reduction in diabetes-related deaths, a 25% reduction in microvascular endpoints [2] and up to a 50% reduction in the risk of microvascular complications and cardiovascular events.[3] However, the translation of evidence-based guidelines in real world settings do not achieve the outcomes seen in highly regulated clinical trial settings.

Indigenous populations globally have significantly high rates of type 2 diabetes compared to their non-Indigenous counterparts. In Australia, the Aboriginal and Torres Strait Islander population is no exception. For many, chronic disease risk factors present early in life and lead to type diabetes in early adulthood. Type 2 diabetes is 7 times more likely in Aboriginal and Torres Strait Islander adults compared to non-Indigenous adults[4] and responsible for a large proportion of premature morbidity and mortality. Of those who have diabetes, many live with a high burden of diabetes complications of the heart and blood vessels, kidneys, eyes and nervous system.[5] Diabetes and its associated complications are directly

attributable to high mortality rates, with Aboriginal and Torres Strait Islander adults being 6 times more likely to die from diabetes than non-Indigenous Australians.[4]

With diabetes primarily managed within the community setting by multiple disciplines, it is essential that local primary health care services have a workforce that can deliver a minimum standard of diabetes care. Public health evidence across low, middle and high-income countries has demonstrated that community health workers are effective in improving population health, particularly where health workforce resources are limited and where health disparities are persistent despite well-developed health systems.[6, 7] Australian examples include, Aboriginal health workers managing diabetes through case management [8] and continuous quality improvement [9] approaches in primary care settings achieving a 1% and 0.4% reduction in HbA1c, respectively. Investing in remote area AHW/Ps can reduce the risk of diabetes-related hospitalisations and can result in hospital cost savings [10].

Diabetes is a specialty field of health care practice. Currently there is not a nationally recognised diabetes course that advances AHW/P knowledge and skills in diabetes. AHW/P in primary care services have completed either a Certificate 3 or 4 which is delivered by a Registered Training Organisation. The diabetes component of this training is based on raising awareness, screening and the provision of healthy lifestyle information and is covered over the course of half a day. The next level of diabetes training for AHW/P is a Graduate Certificate of Diabetes Education and Management that qualifies as initial credentialing by the Australian Diabetes Educators Association. This is followed by 1,000 hours of training required for full credentialing and on-going training to remain credentialed. While this is a specialised diabetes pathway that some AHW/P would be interested in, there is a gap in the career pathway for those who deliver diabetes care and are seeking additional knowledge and best practice support, without becoming credentialed diabetes educators. As AHW/Ps are integral to the multidisciplinary diabetes team in Aboriginal primary care services and permanently available and accessible within the community, a comprehensive understanding and practice of diabetes education and management will serve to strengthen the impact of their role.

There is consistent high level, high quality evidence to indicate that multifaceted interventions which focus on local contexts, address barriers and promote engagement with health care providers are more likely to result in adoption of best practice and hence improved outcomes.[11] A multifaceted knowledge transfer strategy can have positive impacts, as it considers a number of factors which may influence the uptake and implementation of best practice, including but not limited to knowledge, skills, attitudes, personal beliefs and social context. A tailored intervention strategy may utilise a combination of educational meetings, training of health care providers, educational outreach, practice facilitation, local health leaders, peer support and audit and feedback.[11] These interventions, individually and collectively, form the building blocks of knowledge transfer and evidence implementation strategies and have been rigorously evaluated. Given that knowledge, skills, attitudes, personal beliefs and social context of the proposed cohort of health care providers are likely to vary, a multifaceted approach will allow for customisation and development of tailored interventions. Such a nuanced approach is critical as barriers to

evidence-based diabetic care is also multi-factorial and there is no "one-size fits all" when developing these interventions.

The Intervention: Aboriginal Diabetes Workforce Training Program (training program)

The training program consists of 3 components (Figure 1):

1) **Peer Support Network (PSN)**

A state-wide virtual PSN for Aboriginal Health Workers and Practitioners (AHW/Ps).

2) **E-Learning Modules**

Nine self-paced e-Learning modules on the following topics of diabetes care: 1) Introduction; 2) About diabetes; 3) Healthy living; 4) Glucose monitoring; 5) Low and high blood glucose levels; 6) Medicines and insulin; 7) Diabetes-related complications; 8) Support for self-management; 9) Other priority groups

Link to the on-line modules: https://www.ndss.com.au/about-diabetes/aboriginal-and-torres-strait-islander-peoples/health-diabetes-learning/

3) **Onsite Support (OS)**

Practical support for staff to assist with implementing knowledge into practice within their primary care service.

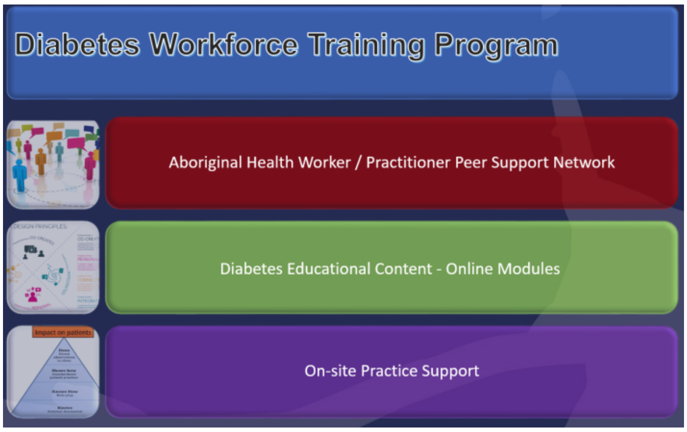

Figure 1: Diabetes workforce training program

3. AIM & OBJECTIVES

Aim

To evaluate the effectiveness of a culturally and contextually informed Aboriginal Diabetes Workforce Training Program (hereafter training program) on Aboriginal primary health care workforce knowledge, attitude, confidence, skill and practice relating to diabetes care.

Objectives

1. To implement the training program in Aboriginal primary health care services across South Australia.
2. To evaluate the effectiveness of the training program on Aboriginal Health Worker, Practitioner and multidisciplinary team knowledge, confidence, skill, practice and

attitude related to diabetes care, and secondary outcomes relating to quality of diabetes care and patient outcomes.

4. HYPOTHESIS

It is hypothesized that the primary health care services whose workforce participate in the training program will have:

- a workforce with increased knowledge, confidence, and skills to manage diabetes within the local primary health care setting,
- in the longer-term, improvements in the quality of diabetes care provided and patient biomedical outcomes.

5. STUDY DESIGN

The study is a Cluster Randomised Crossover trial with two arms (Group A and Group B) (Figure 2). The Cluster Randomised Crossover trial study design ensures that all participating Aboriginal primary health care services across South Australia will have access to the diabetes training, as opposed to only half the services.

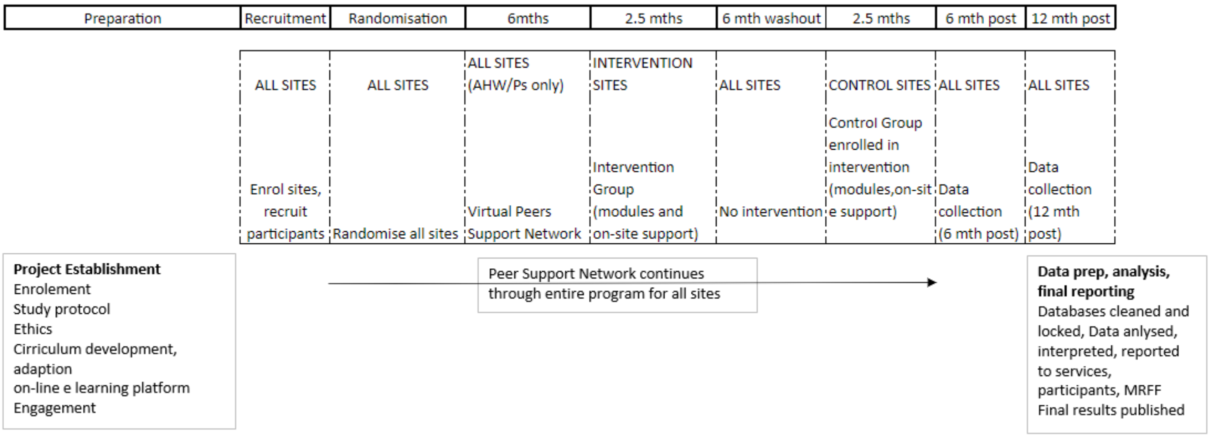

Figure 2: Study design

The research will be initiated and led by Associate Professor Odette Pearson at Wardliparingga Aboriginal Health Equity, South Australian Health and Medical Research Institute (SAHMRI). SAHMRI is South Australia's flagship independent not-for-profit health and medical research institute. SAHMRI's Wardliparingga Aboriginal Health Equity theme is committed to understanding, monitoring, responding to, and reducing inequity in health and wellbeing among Aboriginal and Torres Strait Islander communities. Project coordination will be delivered from SAHMRI North Terrace, Adelaide. The research is funded by the Medical Research Future Fund Primary Health Care Research Initiative.

6. STUDY SETTING

The study will be implemented in the real-world setting of Aboriginal primary health care services in South Australia. These services primarily provide primary health care to Aboriginal and Torres Strait Islander people. Each service is aware of the study.

a. Sites

South Australian Government Health Services are indicated with an asterisk in Table 1.

Table 1: Study sites

Metropolitan	Regional	Remote
Watto Purrunna, (NALHN*)<br><br>Aboriginal Family Clinic, Noarlunga (SALHN*)<br><br>Nunkuwarrin Yunti of SA Inc., Adelaide & Elizabeth Downs	Riverland Community Health Service*<br><br>Yorke and Northern Local Health Network* (Tarpari Wellbeing Centre, Port Pirie & Point Pearce Aboriginal Health Service)<br><br>Moorundi Aboriginal Community Controlled Health Service Inc, Murray Bridge<br><br>Pika Wiya Health Service, Port Augusta<br><br>Port Lincoln Aboriginal Health Service (PLAHS)<br><br>Nunyara Aboriginal Health Service, Whyalla<br><br>Pangula Mannamurna, Mount Gambier	Yadu Health Aboriginal Corporation, Ceduna<br><br>Umoona Tjutagku Health Service, Coober Pedy

*Organisations governed by SA Health

7. STUDY POPULATION AND SETTING

Training program:

The study population will be drawn from the services in Table 1 and includes:

- Aboriginal Health Workers and Practitioners
- Multidisciplinary health care providers

We will attempt to recruit a minimum of two participants from each site.There is no restriction on the maximum number of participants from each service.

Training program enablers and barriers interviews:

A subsample of the training program participants. A purposeful sampling approach will be used to achieve a diversity in AHW/Ps and multi-disciplinary team members, working in urban, rural and remote services.

Service system assessment focus groups:

Chronic disease team members in each service involved in the study. Focus groups with the chronic disease workforce in each participating health services will be conducted to assess the system characteristics for each service.

Medical record review:

Participants will not be recruited; Medical records (existing data) of people with diabetes in each participating service who meet the inclusion criteria will be audited retrospectively.

8. ELIGIBILTY CRITERIA

Inclusion criteria

1. **Training program:** Aboriginal Health Workers/Practitioners and multidisciplinary health care providers are eligible to participate in the study and must meet the following:

Aboriginal Health Worker/Practitioner:

- Completed or currently enrolled in Certificate III/IV in Indigenous Primary Health Care
- Employed by a participating South Australian Aboriginal primary health care service
- Provides diabetes care to Aboriginal clients.

Multidisciplinary health care provider who are:

- From a broad range of service providers, including enrolled and registered nurses, allied health professionals, medical doctors,
- Employed by or provides services in a participating South Australian Aboriginal primary health care service,
- Provides diabetes care to Aboriginal clients.

2. **Interviews for enablers and barriers:** All participants in the training program are eligible to participate in an interview on the enablers and barriers to participation, effectiveness and sustainability of the training program.

3. **Focus group for service systems assessment:** Chronic disease team of health services involved in the project.

4. **Medical record review:** People with diabetes who have attended the participating health service and who fulfil the following criteria at the time of record review:
   - Both male and female who are aged 18 years and over;
   - identify as Aboriginal or Torres and Strait Islander people;
   - have a confirmed diagnosis of T2DM and/or a HbA1c ≥6.5 mmol/L;

- have attended the health service at least twice in the preceding 12-month period;

Exclusion criteria

Training program: Limited or no access to stable Internet connection or computer hardware (laptop, desktop, tablet) can be provided by the health service.

Interviews enablers and barriers: No exclusion criteria

Systems Assessments: No exclusion criteria

Medical record review: No exclusion criteria

9. STUDY OUTCOMES

a. Quantitative

i. Primary Outcome

The primary outcomes of this study relate to diabetes management in a primary care setting that measure participant:

- **Knowledge** measured using the Simplified Diabetes Knowledge Scale – True/False Version [12]
- **Attitude** measured using the Diabetes Attitude Scale (DAS-3) [13].
- **Confidence** measured using the Diabetes Confidence Survey (DCS) [14].
- **Practice and skills** using case studies scenarios that were developed by the research team and the investigators specifically for the project and will be assessed as a composite outcome.
- **Skill** assessed using a case study for a diabetes foot check. This measure was adopted from the Foot Forward Project, which was developed by Twigg, Wischer, and Frank 2021 [15].

ii. Secondary Outcome(s)

The secondary outcomes of this study relate to patient care received and patient clinical outcomes:

- Peer Support Network satisfaction survey
- Interviews of enablers and barriers to participation
- Service systems assessment - Health Service Characteristics: System assessment through conducting focus groups with the chronic disease team staff to assess the system characteristics of each service using the Assessment of Chronic Care Scale (ACIC).
- Medical record review: Quality and Outcomes of diabetes care.

10. STUDY PROCEDURES

a. Recruitment of participants

Training program: All participants will be recruited prior to the commencement of the training program. Only staff employed in sites with a collaborative agreement signed by the service CEO (or delegate) will be approached.

Recruitment of training program participants will be by indirect approach. A manager of the primary health care service will identify eligible staff based on the eligibility criteria. Primary Health Care service CEO's or delegated clinic manager will send an email, drafted by the research team (Attachment *1*) to potential participants who meet the inclusion criteria. The email will include an introduction to the training program, the information sheet and the consent form. Participants will be required to contact the research team to flag their interest in participating in the program. Contact details of the Study Manager are included on the information sheet and in the email.

In addition to individual discussions, the research team will offer to do a group onsite or zoom presentation about the project and what is required from participants. The site manager will invite participants to this and arrange a space for participants to join the zoom meeting or in person. At the group meeting participants will be invited to have an individual discussion about participating in the training program at another time. This will be arranged by a member of the research team and the potential participant. Odette Pearson the project lead will also be involved in the discussion. Potential participants will not be asked to consent to participate in the study at either of these discussions but given time to think about their participation.

Interviews enablers and barriers: All participants will be advised in the training program information sheet and participant consent form that they will be invited to participate in an interview about the barriers and enablers of the training program after they have completed the project. On completing the training program participants will be directly approached by email by a research team member up to three times over 3 weeks to be invited to participate in an interview (Attachment *2*). We will seek to purposefully interview people across geographical locations and disciplines according to the sampling frame below. This will be dependent on participants, for example, there may not be nursing or allied health professionals across all geographical locations.

	Metro	Rural	Remote
Health Worker/Practitioner	1	1	1
Allied Health	1	1	1
Nursing	1	1	1

System assessment: The systems assessment is negotiated in the Collaborative Agreement with each service. If a service agrees to participate in the systems assessment, the CEO (or their nominee) will be provided by the Study Manager a draft email inviting diabetes/chronic disease team members to participate (Attachment *3*). The participant information sheet will be attached. The CEO will arrange a time for the focus group inviting all of those they have identified as eligible. Only those interested in participating will turn up to hear about the study and the purpose of the systems assessment. Focus groups with the chronic disease workforce in each participating health services will be conducted to assess

the system characteristics for each service. There is no upper or lower limit to the number of staff who participate in the focus group; it depends on the staff willing to participate and who are available on the day.

Medical record review: Participants will not be recruited; existing data of people with diabetes in each participating service who meet the inclusion criteria will be audited retrospectively.

Informed consent process

Participation in the Study is voluntary. Several options to ensure potential participants have sufficient information and adequate understanding of the proposed research and the implications for participation will be made available, as follows:

- Participant information sheet
- Presentations on the study by the research team that may be attended by one or more staff from the same service
- Individual phone conversation about what it would mean to participate in the study in the way of purpose, method, demands, risks and potential benefits

With each of these options, potential participants will be invited to have a conversation with a member individually about what participation requires and an opportunity to ask questions.

It will be made clear in all options that not participating will not affect their relationship with their employer or the study team or involvement in future research. A reason for not participating is not required.

It will also be made clear that:

- That the service they are employed by is supportive of the study and has agreed to participation during paid working hours, access to a computer and the internet, on-site support being provided at the service.
- Participants will not be reimbursed for their time spent undertaking the training program
- Data collection is required to evaluate the Diabetes Training Program. We will cover what data will be collected, when including how often, the length of time data collection will take and the format for data collection (e.g., REDCap Survey).
- At any time participants may withdraw from the study without any penalty. The data they have provided can be withdrawn if they wish or can continue to be used in the study with their consent.

Potential participants themselves will have the ability to provide informed consent, no other person or statutory body is required to decide on their participation in the study.

As the methods of the study are pre-defined, full disclosure of the study protocol will be given.

Training program: A member of the research team will meet with each potential participant (either in person or via phone or video call) to explain the details of the study, review the Participant Information Sheet (Attachment *4*), and provide the AHW/P or multidisciplinary health care providers an opportunity to ask the researcher any questions. If potential

participants participate in a team discussion about the project, they will have the opportunity to ask questions and may decide they do not want an additional one on one discussion.

Potential participants will be asked to take their time to think about participating and asked permission to be followed-up in 4-5 days. The research team follow up participants for a maximum of three times, either via email or phone conversation, over three weeks.

If the potential participant does agree to participate in the study, they will be asked to sign a Consent Form (Attachment *5*) and email a copy back to a research team member.

In the event a participant signs the consent form without having discussed the project they will be followed up by the research team to ensure they clearly understand the program and participation. They will be given time to consider their participation, as outlined above.

Interviews enablers and barriers: Once potential participants flag interest in being interviewed, a research team member will discuss the purpose of the interviews, cover the details in the information sheet (Attachment *6*) and what consenting means (Attachment *7*). Potential participants will be given time to decide and be followed up 5 days after initial discussion for a maximum of three times over 3 weeks (Attachment *2*).

Systems assessment: Only those interested in participating will turn up to hear about the study and the purpose of the systems assessment. Prior to starting the focus group, a research team member will go through the participant information sheet (Attachment *8*) and consent form (Attachment *9*) allowing time for questions. If potential participants would like to participate, they will be asked to sign the consent form.

Medical record review: A waiver of consent is being requested to receive medical record data collected in routine clinical practice. These data will provide insight into the effectiveness of the training program on patient outcomes. It is not feasible to ask permission of everyone across multiple services. A similar approach has been used by the research group to develop the SA Aboriginal Diabetes Strategy.

People in dependent or unequal relationships (National Statement Chapter 3.4)

We recognise that employees (AHW/Ps & multidisciplinary health care providers) are in an unequal relationship with their manager.

The site managers will only provide the initial email informing staff of the study and then the research team will recruit participants. During recruitment and on the information sheet voluntary participation, it is clearly stated that non-participation will not jeopardise their employment or opportunities to be involved in further research. This will help mitigate coercion to participate.

To mitigate the potential for managers to prevent their staff from participating in the training program, the research team will first approach the service about the study and seek their support for their staff to participate; if they choose to participate a Collaborative Agreement (Attachment *10*) will be entered into. During the development of the Collaborative Agreement service management will be required to approve staff time to participate and use computers, the internet and zoom facilities; this is to ensure that staff have the necessary resources to be able to participate.

Sample

Training program: We will aim to recruit two participants per service. In discussions about the project with services, most services have more eligible staff. There must be at minimum one participant but is no upper limit to the number of participants from each service. The sample size will include an estimated uptake of (n=40) and accounted for drop-out rate of 50% of the AHW/P. Therefore, (n=20) participants would need to complete the training program.

Interviews enablers and barriers: We will aim to recruit between 3 and 15 participants of the training program given time and feasibility.

Systems assessment: The number of chronic disease staff who participate in the system assessment focus group will vary by service, depending on how large the team is and the number who consent to participate. There is no upper or lower limit to the number of staff who participate in the focus group; it depends on the staff willing to participate and who are available on the day.

Medical record review: Participants will not be recruited; Medical records (existing data) of people with diabetes in each participating service who meet the inclusion criteria will be audited retrospectively. The number of people with diabetes who receive care at each service enrolled in the study and meet the study inclusion criteria will vary. Prevalence of self-reported diabetes varies between 8 and 23% according to the 2018-19 ABS National Health Survey. It is therefore expected that between 8 and 23% of clients at each enrolled service will meet the eligibility criteria.

Time period for recruitment

Training program: Three months is allocated to recruit participants.

Interviews barriers and enablers: Four weeks allocated to recruit participants inclusive of the last two weeks of the training program.

Systems assessment: Services will confirm their participation in the system assessment during the development of the Collaborative Agreement. This will be revisited during the first 12 months of the project at the time we are arranging to do the system assessment. Services can decide then or at any point that they do not want to be involved in the system assessment.

Medical record review: Services will confirm their participation in the medical record review during the development of the Collaborative Agreement. This will be revisited during the first 12 months of the project at the time we are arranging to collect data. Services can decide then or at any point that they do not want to be involved in the medical record review.

b. Study procedures (Figure 3)

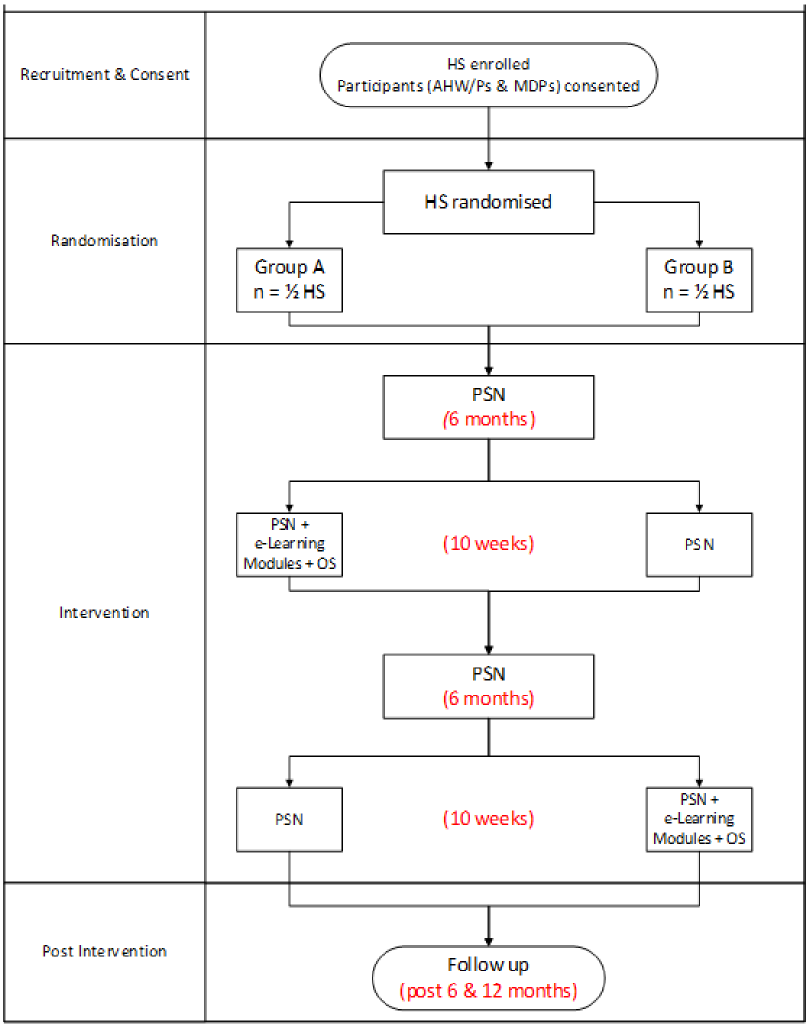

HS = Health Service
AHW/Ps = Aboriginal Health Workers & Practitioners
MDPs = Multidisciplinary health care providers
PSN = Peer Support Network (AHW/Ps only)
OS = Onsite Support

Figure 3: Study procedures flow diagram

Enrolment of study sites

All primary health care services are aware of the study because they were all invited to participate in the co-design of the training program. The Aboriginal Health Council of South Australia advised that ethics approval was not required for the co-design of the training program. Many study sites have identified their interest in participating in the project. A Collaborative Agreement template has been developed (Attachment *10*). The Collaborative Agreement outlines the roles, responsibilities and resource requirements of the research team and the health service, including study timeframes and data collection requirements. Site roles include allowing staff to participate in the project activities during work hours, staff access to internet, computer and zoom facilities, having a staff member who is not a study participant on the study Steering Committee.

Randomisation

Randomisation will occur at the service level. Enrolled health services will be randomised into Group A and Group B. Randomisation and allocation will be undertaken after six months of providing PSN for all enrolled health service sites and prior to commencement of the training program. All enrolled health service sites will be listed, and a statistician will be responsible for randomizing the health service sites into cluster one (Group A participants) or cluster two (Group B participants) using a block size through a computer program for randomisation.

For the intervention assignment; a cluster crossover randomised trial will be employed in this study, as all participants receive the same intervention based on random service site allocation. Participants in the study sites will know which group they have been allocated after completing six months of the PSN, and before commencing the E-Learning module for group A. The project research team will not be blinded to group A or B allocation, only people analyzing the results will be masked/blinded.

Peer Support Network (months 1-17): Only AHW/Ps will participate in the PSN. AHW/P participants from both Group A and Group B will participate in the PSN for the entire duration of the study. The PSN will commence and convene for 6 months prior to Group A doing the e-learning modules and the OS. The PSN will run online via Teams, once a month, for 1.5 to 2 hours duration, and will be facilitated by a credentialled diabetes educator (DNE) who is a research team member.

As this is a pragmatic trial, it is understood that AHW/Ps may not be able to attend all PSN sessions due to competing work and/or personal priorities.

The PSN meetings will not be recorded. The PSN Facilitator will record participation in the network.

E-Learning Modules (10 weeks): There are 9 self-paced e-Learning modules. Each module takes approximately 1 hour to complete. Ten weeks have been allocated to complete the e-learning modules. All participants will do the e-learning modules. Services will have agreed to allow their staff to use the work computers and internet and to do the modules during work hours. These can also be done from any computer, at any time. The modules can be left and picked up at the same place for convenience during working hours.

Onsite Support (minimum of twice over 10-week period): The OS sessions will occur during the same 10-week period as the e-Learning modules. The OS will be face to face at the primary health care service. The number of OS sessions and what is covered during them will be negotiated between the participants and the OS Facilitator. It is suggested that each service hold a minimum of 2 sessions with the OS Facilitator. OS sessions will be held as a group, if there is more than one AHW/P or multidisciplinary health care providers within the health service. The group sessions will be held with AHW/Ps for the first half of the session and then include multidisciplinary health care providers for the remainder of the session. This format will be assessed and adapted as needed. This format responds to direct feedback during the co-design of the training program.

The OS will be facilitated by a credentialled diabetes educator (CDE) employed by Diabetes SA. The CDE will be a member of the research team.

<u>Group A</u>: At the start of month 7, AHW/P and multidisciplinary health care provider participants within the health services randomised to Group A will commence the e-Learning modules and OS, for a duration of 10 weeks. During this time, AHW/Ps from both Group A and Group B will continue to participate in the PSN.

Wash out period (6 months): There is a six month 'wash out' period where only the PSN is running. This will occur between Group A and Group B doing the e-Learning modules and participating in the OS.

<u>Group B</u>: After the 6-month washout period the participating AHW/Ps and multidisciplinary health care providers within the health services randomised to Group B will commence the e-Learning modules and OS, for a duration of 10 weeks. During this time, AHW/Ps from both Group A and Group B will continue to participate in the PSN.

Enabling and supporting on-going connection among the cohort

A Microsoft Teams site will be created by the research team, who will manage the site so that only the research team can invite and remove members. All participants will be invited to the site. It will house diabetes-related resources approved by the study investigators or the Facilitators of the PSN and OS. One folder within the Teams site will be private only for PSN members. This folder will house the PSN materials. The research team and members will be able to post chats on the site to keep in contact. The chats will not be used as data in the study. The site will be administered and monitored by the research team predominantly the PSN Facilitator, OS Facilitator and Study Manager. On completion of the study, participants will be invited to keep the resources and the Teams site will be deleted by the research team.

During the training intervention a weekly email will go out to participants doing the training program to remind and keep participants moving through the modules (Attachment *11*).

On-going communication

A Friday update in the form of an email will be sent to participants (Attachment *12*). This will include the status of the study in the form of a traffic light system and a highlight for the week which will be sourced from a reputable source such as Diabetes Australia, NDSS, RACGP. Each week we will introduce a research team member or a member of the diabetes health profession community including the Investigator team.

An e-newsletter will be produced every 2-months introducing the study, the study investigators, the research team, updates on project progress, links to diabetes resources, diabetes studies and showcasing diabetes organisations will be developed by the research team. It will be distributed to each site through the site representative on the study Steering Committee and to all participants, study investigators and key stakeholders. This will be an important form of communication during the intervention and post intervention. The Principal Researcher, Odette Pearson will be responsible for signing off on the Friday update and the e-newsletter.

Time commitment of participants

Table 2 shows the time commitment by discipline, service, training component and data collection.

Table 2: Time commitment

	Peer Support Network	Modules	Onsite Support	Survey evaluations[a]	Interviews[b]	System Assessment	Medical record review
AHW/P[c]	2 hours/month for 17 months	Maximum 1 hour for each module – 9 modules	Approx. 2-6 hours over 10 weeks	10 minutes x 7 times<br>7 minutes x up to 17 times (PSN)	1 hour once during the study		
Multidisciplinary health care provider	n/a	Maximum 1 hour for each module – 9 modules	Approx. 2-6 hours over 10 weeks	10 minutes x 7 times	1 hour once during the study		
Service						2-3 hours	1 hour each time

[a] Demographics collected three times, will add 5 minutes; collection of primary outcome measures (knowledge, attitude, confidence, practice, skill)

[b] Does not include all participants

[c] Aboriginal Health Worker/Practitioner

Participant withdrawal from the study

If a participant chooses to withdraw from the study before the initiation of data analysis, their data will be separated and will not become part of the study findings. If they chose to withdraw from the study after the data analysis, it will not be possible to retract their data. Any participant wishing to withdraw from the study can contact the research team via the telephone and/or email address provided in the Participant Information Sheet. Friday update and the e-newsletter will also have contact details of the research team and ethics committee approvals. Participants will be asked to complete a participant withdrawal form (Attachment *13*).

c. Methods of data collection

An outline of data collection methods is provided for demographic, primary and secondary outcomes and potential confounding variables. Detailed information including the variables are provided in (Attachment *14*). Table 3 provides a summary of data collection. Data collection procedures are described thereafter.

Data collection timepoints for the primary outcomes

For the entire study there are seven points in time at which data will be collected. These are labelled T0 (baseline), T1 – T6 in Figure 4 and are referred to in the below outline of data collected. Reason for the training program data collection timepoints are as follows:

- T0- Baseline - groups A & B
- T1 - Measure Effect of Peer Support Network - 6 Months Post Baseline Group A & B (Aboriginal Health Workers and Practitioners only).
- T2 – Measure effect of training program (PSN, Online Modules, Onsite Support) on group A - 8.5 Months post-baseline (data collected on both group A & B).
- T3 – Baseline of group B prior to doing the intervention (data collected on both group A & B), 14.5 months post-baseline.
- T4 – Measure effect of training program (PSN, Online Modules, Onsite Support) on group B post group B undertaking the intervention at 17 months from baseline (data collected on both group A & B).
- T5 – Sustainability of effect of training program on group A 23 months post-baseline, and group B 6 months post commencement of intervention.
- T6 - Sustainability of effect of training program on group A 29 months post-baseline, and group B 12 months post commencement of intervention.

Data collection timepoints for secondary outcomes

1. Peer Support Network satisfaction survey: This will be collected monthly after each Peer Support Network Meeting for a total of 29 months post-baseline.
2. Interviews of enablers and barriers to participation: this will be undertaken during:
    - T2 with Group A (8.5 Months post-baseline), and
    - T4 with Group B (post group B undertaking the intervention at 17 months from baseline).
3. Medical record review: Quality and Outcomes of diabetes care: This will be undertaken during:
   T1 - (6 Months Post Baseline Group A & B)
   T4 - Measure effect of training program (PSN, Online Modules, Onsite Support) on group B post group B undertaking the intervention at 17 months from baseline (data collected on both group A & B).
   T6 - Sustainability of effect of training program on group A 29 months post-baseline, and group B 12 months post commencement of intervention.
4. Service systems assessment - Health Service Characteristics (through focus groups): This will be undertaken once during the study between months 3 and 12 from the start of the study.

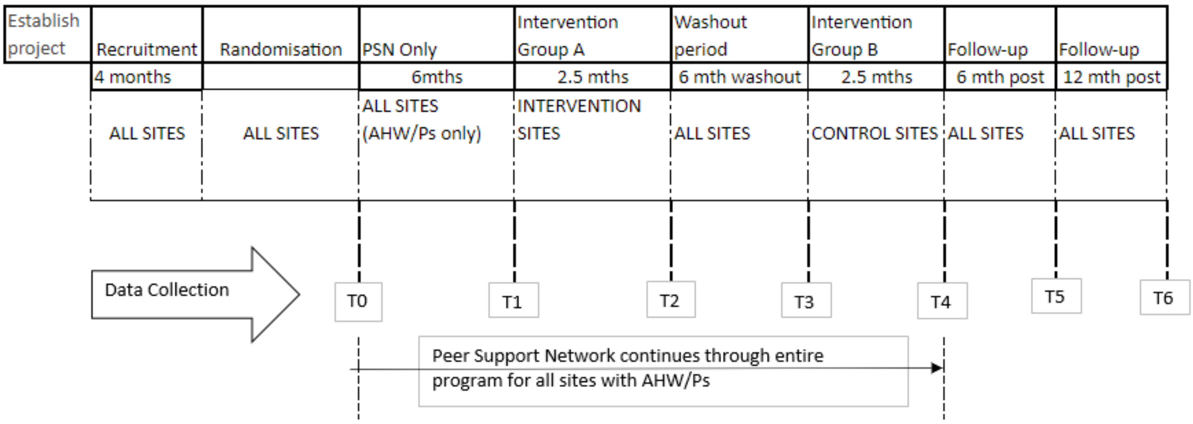

Figure 4: Data collection timepoints

Category	Description	Validated tool	Number of variables	When or Timeframe	How	From whom
Demographics	Characteristics of individuals, their work experience and the site they are employed by	Research team developed	11	T0, T5, T6	RedCap	All participants
Primary Outcomes	Diabetes knowledge	Simplified diabetes knowledge scale	20	T0, T1, T2, T3, T4, T5, T6	RedCap	All participants
	Confidence in managing diabetes	Diabetes Confidence Survey	15	T0, T1, T2, T3, T4, T5, T6	RedCap	All participants
	Attitude relating to diabetes	Diabetes Attitude Scale (DAS-3)	25	T0, T1, T2, T3, T4, T5, T6	RedCap	All participants
	Diabetes management in practice & skill	Research team developed	9	T0, T1, T2, T3, T4, T5, T6	RedCap	All participants
	Diabetes management skillm (foot check)	Research team developed	10	T0, T1, T2, T3, T4, T5, T6	RedCap	All participants
Secondary Outcomes	Evaluation of Peer Support Network	Research team developed	9	Monthly after each PSN	RedCap	Aboriginal Health Workers and Practitioners
	Enablers and barriers of participation, sustainability and effectiveness of the training program	Research team developed	up to 12	Once during the study at T2 with Group A and T4 with Group B	Semi-structured interveiw - face to face or zoom	A cross section of participants from each discipline; 3 to 5 with a total of up to 15 participants
	Diabetes patient behaviour and clinical outcomes	Assessed against RACGP evidence-based care guidelines	up to 8	Jan 2018 - 12 month post training intervention (T1, T4, T6)	Retrospective electronic data extraction	Electronic medical records of clients with diabetes in each site
	Quality of diabetes care	Assessed against RACGP evidence-based care guidelines	up to 35	Jan 2018 - 12 month post training intervention	Retrospective electronic data extraction	Electronic medical records of clients with diabetes
Potential confounding variables	Variables that have the potential to reduce bias in statistical models to give a more accurate estimate of cause and effect of the training program	Based on Directed Acyclical Graphs	5	Varied	RedCap (3 variables are collected in demographic and PSN evaluation)	All participants
Health Service Characteristics	Health service characteristics for delivery of evidence based diabetes care	Assessment of Chronic Care Scale	up to 50	Once between months 3 and 12 of the study commencement	Face to face or zoom	Multi-D team diabetes or chronic disease team within each service

Table 3: Summary of data collection methods

Data collection procedures

Demographics, primary outcomes, potential confounding and peer support network evaluation variables

At each relevant time point of data collection, each participant will receive an email from the Study Manager with a unique URL link to the RedCap Survey. Each time a survey is sent, participants will have up to 14 days to complete the survey. For incomplete surveys, up to three email prompts and one phone call will be used to remind participants to complete the survey. At each data collection time point survey's will be closed after 14 days.

Demographics are collected three times during the study period and will take 5 minutes to complete. The primary outcomes are collected seven times and in total take up to 35 minutes to complete. The peer support network evaluation questions are collected monthly after each PSN session and take approximately 5 minutes to complete.

Surveys can be programmed to be sent automatically on a given date which can be changed as well as sent manually if required.

Interviews enablers and barriers of participation, sustainability and effectiveness of the training program

Dr Sana Ishaque, an experienced qualitative researcher with a medical background, with support from a practicing Aboriginal Health Practitioner will conduct the interviews. Interviews will be conducted face to face or by Zoom. If face to face, participants will be asked if they could arrange a space within their health service or if they would like the study team to arrange a space in public area such as a room in a local library or in another organisation. While all participants will be invited to participate in an interview, we will aim for a maximum of 15 participants given time and feasibility. If there is a choice (based on recruitment), we aim to have representation across geographic locations and service sizes and systems infrastructure, with a mix of AHW/Ps, Aboriginal nurses, and multidisciplinary staff. Participants will be asked permission for the interviews to be recorded. Interviews will be transcribed professionally, and participants will be invited to add or edit their transcript.

System assessment

Sites vary in the systems and infrastructure available to enable and support the management of type 2 diabetes. The importance of assessing these service characteristics and taking them into consideration in interpreting study findings was emphasised in discussions with the Aboriginal Health Council of South Australia in the development of the grant proposal.

Focus groups with the chronic disease team or staff will be conducted to assess the system characteristics of each service. These will be conducted face to face or via zoom. Focus groups will occur at a time suitable to the service, within the first 12 months of trial commencement. Two researchers will conduct the focus groups. The ACIC will be used to inform the focus of discussions and system assessment. Participants will be provided with the assessment scale prior to the focus group. The focus groups will not be recorded. Staff who participated will be given the opportunity to review the assessment and provide feedback which will be added.

Medical record review (quality of diabetes care and diabetes clinical outcomes)

Negotiating collaborative agreements with sites prior to study implementation includes the sites agreeing or not agreeing to provide de-identified data from their electronic patient management system (PMS) on their diabetes clients, as defined in section 10.d. As the sites do not all use the same type of PMS, the study will work with the clinic/data manager at each site to determine the best data extraction method for their individual electronic PMS. This will likely require a data extraction report to be written (coded) by the data manager from the site or by the PMS vendor (e.g., Medical Director, Communicare). Data extraction software may also be utilised, such as CAT Plus from PENCS, which compatible with the majority of PMS across Australia. All identifiable data extracted from a PMS using CAT Plus is securely stored within the service and not moved offsite.

The data extraction will be run onsite by an appropriate staff member (as identified by the health service) and saved as a CSV file. The extracted data will be de-identified. The CSV file will be password protected and sent by secure email to the Study Manager.

The study will support the services with the data extraction, as required, including providing reimbursement for their time.

The number of times electronic medical record data is collected will be negotiated with each individual service - ideally, at the beginning, midway and at the end of the project (namely, at T1, T4 and T6(Figure 4).

d. Access to existing data

Name/Description of data	Electronic Medical Records
**Data Custodian**	SA Health Primary Health Care Sites - Watto Purrunna (Muna Paiendi, Wonggangga Turtpandi), Aboriginal Family Clinic (Noarlunga, Clovelly Park), Yorke & Northern LHN (Point Pearce Port Pirie), Riverland Mallee Coorong LHN.
**Agency Type**	State government Aboriginal primary care service
**Data Collection Format**	De-identifiable
Variable	***Justification***
Health service level	***To provide a basic description / service context for each service.***
Number of regular clients	*Defined as 3 or more visits in the previous 2 years. This will become the denominator for the proportion of clients with diabetes within a service.*
Number of regular and transient clients on a care plan	*The number of current/regular and the number of transient Aboriginal clients, registered with the following conditions, including the number in each category and disease group with a current disease-specific care plan.*<br>• *Cardiovascular Disease*<br>• *Renal Disease*<br>• *Mental Health*<br>• *Diabetes*
Number of transient clients	*The number of clients who are defined as a transient client receiving care at the health service. Transience defined as < 2 visits in 12 months.*
Indigenous status	*Proportion of Aboriginal clients using the service of total client population*
Episodes of care	*The number of episodes of care received by Aboriginal clients in the previous 12 months. An episode of care is defined as a presentation to the health service where a service was provided.*
Client level	Eligibility criteria: People with diabetes who have attended the participating health service and who fulfil the following criteria at the time of record review meet the following criteria:<br>• Both males and females who aged 18 years and over;<br>• identify as Aboriginal or Torres and Strait Islander people;<br>• have a confirmed diagnosis of T2DM and/or a HbA1c ≥6.5 mmol/L;<br>• have attended the health service at least twice in the preceding 12-month period;<br>Those who meet the eligibility criteria become the study population in which the below variables are extracted.
Client ID	Service unique record ID created and provided by the health service so that no identifying data is provided to the study team
Month & Year of Birth	To estimate age

Sex	To examine gender differences
Ethnicity	To identify Aboriginal status
GP Management Plan	To calculate number of clients who have a GPMP
Team Care Arrangements	To calculate number of clients who have a TCA
GP Management Plan Review	To identify proportion who have had a GPMP review within the recommended timeframe according to evidence-based care guidelines
Diabetic retinal check performed	To identify proportion who have had a GPMP review within the recommended timeframe according to evidence-based care guidelines
Diabetic foot check performed	To identify proportion who have had a foot review within the recommended timeframe according to evidence-based care guidelines
Absolute CV risk assessment	To identify proportion who have had a CV risk assessment within the recommended timeframe according to evidence-based care guidelines
BMI	Identify proportion within recommended range
Waist circumference	Identify proportion within recommended range
Smoking status	Understand proportion of clients with behavioural risk factors
Physical activity	Understand proportion of clients with behavioural risk factors
Influenza vaccination up to date	Vaccination status
Pneumococcal vaccination up to date	Vaccination status
Covid-19 vaccination	Vaccination status
Clinical measurements:<br>– *Cholesterol (mmol/L)*<br>– *LDL (mmol/L)*<br>– *HDL (mmol/L)*<br>– *Triglycerides (mmol/L)*<br>– *Creatinine (μmol/L)*<br>– *eGFR (ml/min/1.73m²)*<br>– *uACR (mg/mmol)*<br>– *Random BGL (mmol/L) - non-fasting, blood glucose test performed at the health service*<br>– *HbA1c (mmol/mol) HbA1c (%)*<br>– *Systolic BPM (mm/Hg)*<br>– *Diastolic BPM (mm/Hg)*	Identify proportion within recommended range
Number of failed follow up attempts in past 12 months	Service utilisation

Related conditions of clients with diabetes:<br>– *Coronary heart disease*<br>– *Stroke or TIA*<br>– *Peripheral artery disease*<br>– *Congestive heart failure*<br>– *Chronic kidney disease*<br>– *Depression*<br>– *Hypertension*<br>– *Dyslipidaema*<br>– *Cardiac dysrhythmia*	Understand burden of chronic disease among clients.
Referrals to diabetes educator, diabetes specialist, optometrist, podiatrist, dietician, dentist, psychologist, social worker, exercise physiologist, mental health screening	Understand quality of care referral

e. Data linkage

Not applicable.

f. Safety considerations

Potential risks of participating in the research and strategies to mitigate and manage them are outlined below. It is anticipated that any risks to participants will be minimal and limited to discomfort or inconvenience, as defined by the *National Statement section 2.1*. The benefits to participating in the research are believed to justify any potential risks. All risks will be brought to the attention of the Principal Investigator by the research team member/s and/or a participant/s and/or steering committee member. The Principal Investigator will assess if the risk needs to be elevated to the research investigators based on its severity. A risk register will be kept that identifies the risk and severity of the risk, who was affected (not name but if they were a participant, service manager, etc.), who the risk was reported to, the immediate action and how the same risk in the future will be minimized or mitigated. It is the responsibility of the individual who raises the risk with the Principal Investigator to complete the risk register or the Principal Investigator if a participant or someone other than a study team member. The risk register will be included in the papers of each Investigator meeting.

It is possible that participants may be inconvenienced by the work and/or personal time required to participate in the research. This potential burden has been considered in the design of the training program, by ensuring that the program is flexible and obtaining support from service management as an eligibility to be in the study.

Obtaining consent from participants to check on their wellbeing will be discussed in the consent process and is included in consent forms and the distress protocols.

Peer Support Network sessions: Differences in opinions/ideas may cause conflict between participants, which could lead to participants feeling upset or uncomfortable in contributing to discussion. In the event this occurs, the PSN Facilitator will follow the PSN Distress Protocol (Attachment *15*), which may include pausing or terminating the session. The PSN Facilitator will notify the Principal Investigator of the incident and document the incident. A follow up wellness check will be conducted with the affected participant(s). If conflict arises between the PSN Facilitator and participating AHW/Ps during the online PSN sessions, the PSN Facilitator will terminate the session and contact the Principal Investigator for support.

Onsite Support: Professional/personal differences may arise between the participants and the OS Facilitator. If this occurs, participants can contact a member of the research team as indicated in the Participant Information Sheet. Any risks that arise in the provision of routine client care should be dealt with according the policies in place at the participant's health service. A distress protocol will be followed if distress should arise (Attachment *16*).

Interviews enablers and barriers: In the unlikely event that participants become upset while reflecting on their experience of the education program, the interviewer will follow the Individual Interview Distress Protocol (Attachment *17*).

Systems assessment: Differences in opinions/ideas may cause conflict between participants, which could lead to participants feeling upset or uncomfortable in contributing to discussion. In the event this occurs, the Facilitator will follow a distress protocol (Attachment *18*), which may include pausing or terminating the session. The Facilitator will notify the Principal Investigator of the incident and document the incident. A follow up wellness check will be conducted with the affected participant(s). If conflict arises between the Facilitator and participating staff during the focus group, the Facilitator will terminate the session and contact the Principal Investigator and Service Manager for support.

g. Data monitoring

The Study Manager, with guidance and support of the Principal Investigator, Odette Pearson will manage the data. All data will be entered into a computerised database with regular automated backups. Checks for accuracy and completeness will be done shortly after data collection. Computer codes and site randomisation schedule will be restricted to the Study Manager and Principal Investigator and maintained in a secure server unavailable to the data analyst.

Status of survey completion for each participant can be seen in a separate part of the RedCap survey that is not connected to individual survey responses. This shows whether the survey has been completed, started but not completed and not yet started. RedCap capability allows for reminders with survey links to be sent to individual participants yet to complete the survey.

For the research documentation, the PSN and OS Facilitators will keep a reflective journal that will describe the session from their perspective, barriers and enablers to successful

uptake and impact and what could be improved. As the Facilitators are research team members, on advice from the HREC sub-committee, they are not considered research participants and their documentation can be used to inform improvements of the study and be used to describe and evaluate the study. They will record the date of the session and document:

- Detailed description of what was done during each session
- From the Facilitators' perspective, what went well in the session and what could have been done better and potential mitigation or strategies for improvement.
- They will be asked not to record individual or identifying information of individual participants.

These will be collected within 5 days of each session by the Study Manager.

h. Protocol deviations

The Principal Investigator with all Investigators will monitor protocol deviations and/or adverse events. These will be reported to the SA Department for Health and Wellbeing HREC and site-specific research governance officers within 72 hours of identification of the event. Corrective actions will be implemented promptly.

i. Unexpected or serious adverse events

The Principal Investigator will use continuous vigilance to identify and report adverse events within 72 hours of identification of the event to all approving HRECs and relevant Research Governance Officers.

11. DATA ANALYSIS

a. Quantitative

Statistical methods

Dr Gloria Mejia, epidemiologist and Chief Investigator on the project and has led the development of the statistical plan.

Primary outcomes

Directed Acyclic Graphs (DAGs) are being used to determine variables that need to be collected to adjust for potential confounding within statistical models. DAGs enable the conceptualisation of causal pathways. A DAG has been done for each primary outcome.

Generalized linear mixed effects models (GLMMs) with compound symmetric (exchangeable) correlation structure will be used to account for within health care centre (cluster) correlation; logit or log link will be used for binary outcomes such as prevalence resulting in mixed effect logistic regression, while log link will be used for count outcomes resulting in mixed effects negative binomial regression. GLMMs are a missing data method only assuming missing at random – MAR (instead of the more stringent MCAR – missing

completely at random). Effect measures will also be presented through calculation of number needed to treat and its associated 95% confidence interval. Analysis after the 12-month intervention follow-up is planned, spending alpha=0.005.

In addition, a descriptive pre-post analysis of survey results due to participating in the PSN alone in the first six months will be calculated using appropriate parametric or non-parametric tests. These analyses will occur after each collection timepoint.

Secondary Outcomes

Provision of evidence-based care and patient biomedical outcomes will be summarised using counts, means and percentages and differences between time collection points reported. Patients will be stratified by for example, age, gender, diabetes type and existing comorbidities.

b. Qualitative

The qualitative data from the study will be analysed using a data-driven, inductive, thematic analysis based on the Braun and Clarke's guide for thematic analysis. Inductive analysis is a process of data coding without fitting it into any pre-existing coding framework. The majority of qualitative data for the study will come from the interviews. The interview transcripts will be audio recorded and transcribed verbatim. The analysis will begin by the analyst repeatedly reading and re-reading the data to become familiar with it. The more formal coding will start once the analyst becomes familiarised with the data. Once the data has been coded, these codes will be sorted into potential themes and subthemes. Once the themes are reviewed, refined, and finalised they will be used to write the final report. We will aim to analyse and report the data separately for AHW and multi-disciplinary team. Combining all disciplines, we will aim to analyse the data by metro and remote.

b. De-identification

Data will be collected at several points in time.

Individual level data – training program participants: On entry into the study each participant will be allocated a unique ID (URID) within RedCap. The Study Manager will enter enrolled participant details into RedCap including participant first name, surname, date of birth, email address, enrolment date, consent status, consent status and consent form. This will create a participant list and will assign each participant a URID within RedCap, so that their baseline and subsequent evaluations can be linked for the duration of the study. REDCap keeps the URID, and participant name separate to the participant data. The participant data will only include the URID. Only de-identified data will be analysed during the study. Individual data will not become part of any report or publication of the project.

Data collected by semi-structured interviews with AHW/Ps and multidisciplinary health care providers will not be linked to the survey data. This data will be aggregated and thematically analysed. Name or date of birth will not be collected from interview participants.

Service level data (systems assessment): Each health service will be given a unique ID by the Study Manager. A linking key that holds the service name and unique service ID will be kept in a separate file location. All service level information will include the unique ID only.

Quality of care and patient outcomes (medical record review): The health service will allocate a unique ID to each patient record, and this will be kept by the service. Only de-identified audit data will be provided to the research team (Attachment 14). Should the research team need to clarify anything, they will provide the relevant health service with the unique ID number; the health service will then follow-up the query using the patient record.

12. DATA HANDLING AND RECORD KEEPING

a. Data collection and management responsibilities

Data collection is the responsibility of the research staff of Wardliparingga Aboriginal Health Equity, SAHMRI, under the supervision of the Principal Investigator. The Data Manager will oversee data management under supervision of the Principal Investigator. The Principal Investigator is responsible for ensuring the accuracy, completeness, legibility, and timeliness of the data reported. The SAHMRI IT department has the expertise and resources to support the management of research data to levels required by external data custodians and as outlined in the National Statement.

Electronic data will be stored on servers protected by firewalls, security groups and passwords. Data will be stored on the secure S Drive. Accessing the S Drive requires a two-step authentication process that comprises of a SAHMRI log in and then individual access to the S Drive folder arranged by the SAHMRI IT. SAHMRI IT requires evidence of ethics approval for those individuals who request access to the study folder located on S Drive.

Data are protected in accordance with the data management policies of Wardliparingga Aboriginal Health Equity, SAHMRI.

b. Study records retention

All data will be saved in electronic format on password-protected computers in a locked office of Aboriginal Health Equity, Level 4, North Terrace, SAHMRI. On S Drive one master folder will be created that will hold two folders for: 1) individual level data demographics, primary outcomes, potential confounding and peer support network evaluation variables and enablers and barrier to participation, sustainability and effectiveness, and 2) service level data quality and outcomes of diabetes care and service characteristics.

RedCap is an electronic data capture program that will be used to capture most data in this project. RedCap sits on a secure server in Australia that is managed and controlled by SAHMRI. The RedCap unique ID and participant name is stored separately to the participant data which will only record the unique ID. Only the unique ID can be seen and extracted by the research team.

Hardcopy data will be scanned and saved on the S Drive. Audio-transcriptions will be saved on the S Drive.

The Principal Investigator is responsible for retaining data. All data will be stored for 7 years, after which time it will be destroyed in accordance with SAHMRI policy and procedures. Hardcopy data will be destroyed once this is completed at the first opportunity after data collection. Once semi-structured interview data has been transcribed, the audio recording will be destroyed.

13. PUBLICATION & INTELLECTUAL PROPERTY

Data from participating Aboriginal primary health care services and individual multidisciplinary health care providers will remain theirs to own, protect, and control. Interview transcripts will be provided to individual participants to review and to keep, should they wish to. System assessment and analysed audit data will be provided to the individual health service that it belongs to. Only aggregated data will be used to generate study outputs. Wardliparingga Aboriginal Health Equity, SAHMRI, will own the Intellectual Property from the study.

Publication of study findings will include:

- Health service reports and presentations
- Key stakeholder information sheets that provide the study findings
- Conference presentations
- Peer reviewed journal publications describing results from each stage

A knowledge exchange and translation plan will be developed with the Steering Committee.

a. Dissemination of results to participants

Training program evaluation: Where able, participating AHW/Ps and Aboriginal primary care health services will receive results as they are produced.

Peer Support Network: Each month, aggregated results of the survey evaluation will be provided to the Peers Support Network and used to improve the Network.

Systems assessment: Each service will receive their systems assessment within 3 months of it being completed.

Results will be disseminated via email or via the Teams site in the case of the Peers Support Network evaluation and communicated through the Steering Committee.

Key stakeholders will be provided study results, they include National Association of Aboriginal and Torres Strait Islander Health Workers and Practitioners, Diabetes Australia and their state and territory affiliate members, the National Association of Diabetes Centres, Australian Diabetes Educator Association and the National Aboriginal Community Controlled Health Organisation (NACCHO). As host of the e-learning modules, Diabetes Queensland will be provided aggregated results (only those published) of the evaluations built into the e-Learning modules to inform continual improvements of the e-Learning modules.

Medical record review: Each health service will be offered aggregated analysed service level data on patients of their service only. All services in the study will receive combined aggregated results of the audits as they become available.

14. ETHICAL CONSIDERATIONS

The aim is that participants will receive direct benefit from doing the training program in the form of increased knowledge, confidence, skill, practice and attitude towards managing diabetes. Aboriginal Health Workers and Practitioners will benefit from being involved in the Peer Support Network through increased collegial support, sharing information and experiences, and having access to a diabetes nurse education facilitator. All health professionals involved will have access to the e-learning modules for free, they are currently available now for free, hosted on the Diabetes Queensland website. On completion all participants will receive 10 continuing professional development points and a certificate. Participants will also have access to a diabetes educator who will facilitate the onsite support. Only employees within services that allow their staff to participate and use the service internet, computer and clinic site are invited to participate. Benefit to clients with regards to improved quality of care and care outcomes will be evaluated, however the study will not interact with clients, and has requested a waiver to access their diabetes related data to measure potential changes in outcomes.

a. Indemnity & compensation for injury

SAHMRI will work with several sites to deliver the project. Each site participating in the study employs participants within the study and carries appropriate insurance.

b. Vulnerable populations

The study includes Aboriginal and Torres Strait Islander Health Professionals as participants in the training program. The existing Certificate III and IV in Aboriginal Primary Health Care, spends less than one day on diabetes specifically and approximately 2 weeks on chronic disease. As a result, consultations with the workforce identified the need for more training in diabetes management but also less than having to do a diploma or credentialled diabetes educator qualification. The study has been designed for and by Aboriginal Health Workers and Practitioners, importantly the content and practical activities are within their scope of practice.

This study has the potential to inform future training and is of interest to professional bodies. Aboriginal Health Workers and Practitioners have pivotal roles in diabetes management within the community health service and should be supported to provide the best care possible within their practice. Training and a professional network were identified during community consultations in the development of the SA Aboriginal Diabetes Strategy.

c. Waiver of consent

A waiver of consent is being requested to access pre-existing, de-identified patient information to perform the medical record review. The data requested is collected as part of routine client care. Data will only be re-identifiable by the health service that provided the de-

identified data. Justification for a waiver of consent under section 2.3.10 (a-i) of the National Statement on Ethical Conduct in Human Research is provided as follows:

a) involvement in the research carries no more than low risk (see paragraphs 2.1.6 and 2.1.7, page 18 of the National Statement) to participants

The use of patient data in the context of this study carries low risk. Risk is associated with maintaining data security and anonymity, for which the project has strict control measures and monitoring in place. The use of patient data in the context of this study is to determine the effectiveness of the training program on quality of care and patient outcomes. To further protect the identity of individual clients, the data of all clients will be collected and analysed in aggregate at the service level as opposed to individual outcomes.

b) the benefits from the research justify any risks of harm associated with not seeking consent

Aboriginal Health Workers and Practitioners have typically participated in countless hours of training across a wide range of areas. Most training is not evaluated for effectiveness. The program logic of this study is that effective training in diabetes management leads to improvements in evidence-based knowledge, skills and practice and positive attitudes of diabetes which in turn leads to improved quality of care and patient outcomes. It is important that where possible such end points are evaluated to understand future investment of health workers and training providers in time and resources and critically the benefit to clients.

c) it is impracticable to obtain consent (for example, due to the quantity, age or accessibility of records)

The high prevalence of diabetes within the Aboriginal population means that from 7% up to 30% of clients will have diabetes across the potential 17 health services. It would be impractical to obtain consent from this large number of clients.

d) there is no known or likely reason for thinking that participants would not have consented if they had been asked

During consultations for the SA Diabetes Strategies, the Aboriginal workforce was identified as enabler, including the establishment of a peer support network and culturally and contextually appropriate diabetes training. The collection of this data is to evaluate the effectiveness of this training initiative, a direct response to the community consultations. There is no reason for thinking that clients would not allow their data to be used for this purpose. We have experienced this for a previous study. A waiver of consent to collect audit data as part of the Diabetes study was provided in 2014.

e) there is sufficient protection of their privacy

Refer to section 11.c. De-identification.

f) there is an adequate plan to protect the confidentiality of data

Refer to section 12. Data handling and recording keeping.

g) in case the results have significance for the participants' welfare there is, where practicable, a plan for making information arising from the research available to them (for example, via a disease-specific website or regional news media)

The context of this study is not for clinical review.

h) the possibility of commercial exploitation of derivatives of the data or tissue will not deprive the participants of any financial benefits to which they would be entitled

There is no commercial interest.

i) the waiver is not prohibited by State, federal, or international law.

The waiver is not known to be prohibited by State, federal, or international law.

d. Confidentiality

There are multiple methods of data collection employed in this trial. The types of data to be collected from the AHW/Ps include their demographics, and outcome questionnaires to assess their knowledge, confidence, attitude, skills, and practice. This data will be collected via online RedCap surveys. Only the individual who receives the email link to the RedCap survey can access the survey. Submitted RedCap surveys cannot be accessed by participants at any time through the survey link.

A subgroup of participants will be interviewed. These interviews will be audio recorded and later transcribed. The RedCap data and the interview data will be de-identified and will be saved on password protected computers in the office of Aboriginal Health Equity, SAHMRI according to the data retention policy.

Data collected in the study will only be accessed by the research team involved in this trial.

e. Ethical review

The study will be conducted in full conformance with principles of the "Declaration of Helsinki", Good Clinical Practice (GCP), the National Statement on Ethical Conduct in Human Research (NHMRC, 2007), Australian Code for the Responsible Conduct of Research (2007) and within the laws and regulations Australia. As well as, National Health and Medical Research Council, Ethical conduct in research with Aboriginal and Torres Strait Islander Peoples and communities: Guidelines for researchers and stakeholders (2018), Commonwealth of Australia: Canberra and guided by, National Health and Medical Research Council, Keeping research on track II: A companion document to Ethical conduct in research with Aboriginal and Torres Strait Islander Peoples and communities: Guidelines for researchers and stakeholders (2018), Commonwealth of Australia: Canberra.

Ethical approval will be sought from the following HRECs:

- SA Aboriginal Health Research Ethics Committee (AHREC)
- SA Department for Health and Wellbeing Human Research Ethics Committee

f. Site/Governance Review

In accordance with the *SA Health Research Governance Policy Directive,* Site Specific Assessment (SSA) Approval will be sought from individual public health sites where the project is being conducted.

The study Investigator Group will continue to meet for one hour every 6 weeks to oversee the research conduct and rigor.

A project Steering Committee comprising of one representative from each service who is not a study participant will be convened for one hour every two months to oversee the implementation of the study.

15. OUTCOMES AND SIGNIFICANCE

This study presents a unique opportunity to implement a training program for multiple disciplines working in Aboriginal primary health care services and providing diabetes care to Aboriginal clients. With statewide participation of health professionals, the training program has the ability to create baseline knowledge, attitude, practice, skill and confidence with regards to managing type 2 diabetes across primary health care services in SA. As diabetes is so prevalent within the Aboriginal community, having a minimum standard of care provided can benefit those with diabetes and their families.

16. REFERENCES

1. World Health Organization, *World Health Statistics 2018 : monitoring health for the SDGs : sustainable development goals*. 2018, Geneva: World Health Organization.

2. *Intensive blood-glucose control with sulphonylureas or insulin compared with conventional treatment and risk of complications in patients with type 2 diabetes (UKPDS 33).* The Lancet (British edition), 1998. **352**(9131): p. 837-853.

3. Gæde, P., et al., *Multifactorial Intervention and Cardiovascular Disease in Patients with Type 2 Diabetes.* The New England journal of medicine, 2003. **348**(5): p. 383-393.

4. Australian Institute of Health and Welfare, *The health and welfare of Australia's Aboriginal and Torres Strait Islander peoples: 2015*. 2015, AIHW: Canberra.

5. Australian Institute of Health and Welfare, *The Health and Welfare of Australia's Aboriginal and Torres Strait Islander Peoples: 2008*. 2008, AIHW: Canberra.

6. Perry, H.B., R. Zulliger, and M.M. Rogers, *Community health workers in low-, middle-, and high-income countries: an overview of their history, recent evolution, and current effectiveness.* Annual review of public health, 2014. **35**(1): p. 399-421.

7. Chang, A.B., et al., *Indigenous healthcare worker involvement for Indigenous adults and children with asthma.* Cochrane database of systematic reviews, 2007. **2011**(7).

8. McDermott, R.A., et al., *Community health workers improve diabetes care in remote Australian Indigenous communities: results of a pragmatic cluster randomized controlled trial.* BMC health services research, 2015. **15**(1): p. 68.

9. Bailie, R., et al., *Improving organisational systems for diabetes care in Australian Indigenous communities.* BMC health services research, 2007. **7**(1): p. 67.

10. Gibson, O., *The impact of primary health care on hospitalisation of Aboriginal and Torres Strait Islander adults with type 2 diabetes in far north Queensland, Australia*. 2013, University of South Australia,: Adelaide.

11. Pantoja, T., et al., *Implementation strategies for health systems in low-income countries: an overview of systematic reviews.* Cochrane database of systematic reviews, 2017. **2017**(9).

12. Collins, G.S., et al., *Short Report: Education and Psychological Aspects Modification and validation of the Revised Diabetes Knowledge Scale.* Diabetic medicine, 2011. **28**(3): p. 306-310.

13. Anderson, R.M., et al., *The third version of the Diabetes Attitude Scale.* Diabetes care, 1998. **21**(9): p. 1403-1407.

14. Colleran, K., et al., *Building Capacity to Reduce Disparities in Diabetes: Training Community Health Workers Using an Integrated Distance Learning Model.* The Diabetes educator, 2012. **38**(3): p. 386-396.

15. National Diabetes Services Scheme. *FootForward for Diabetes Management*. 2021; Available from: https://nadc.net.au/footforward/publications/.

16. Braun, V. and V. Clarke, *Using thematic analysis in psychology.* Qualitative research in psychology, 2006. **3**(2): p. 77-101.
