# Supplementary material for: Translation of culturally and contextually informed diabetes training for Aboriginal primary health care providers on Aboriginal client outcomes: Protocol of a cluster randomized crossover trial of effectiveness
Source: PLoS One. 2024 Jul 23;19(7):e0305472. doi: 10.1371/journal.pone.0305472 (PMC11265707; doi:10.1371/journal.pone.0305472)
Supplement: Attachment — Submitted filename: Authors response to reviewers feedback.docx [file pone.0305472.s010.docx]

| **Feedback items** | **Authors response (Amended/added)** | **Page and line number** |
| --- | --- | --- |
| **Journal requirements (Editor feedback)** | | |
| 1. **Please ensure that your manuscript meets PLOS ONE's style requirements, including those for file naming. The PLOS ONE style templates can be found at**  <https://journals.plos.org/plosone/s/file?id=wjVg/PLOSOne_formatting_sample_main_body.pdf> and  <https://journals.plos.org/plosone/s/file?id=ba62/PLOSOne_formatting_sample_title_authors_affiliations.pdf> | Thank you for your feedback; the manuscript has been checked and amended, including file naming and formatting of authors’ affiliations. | Pages1-2, 6, 11-12, 16, 20 and 29 |
| **2.** Thank you for stating the following in the Competing Interests section:  "Odette Pearson, Alex Brown, David Jesudason, Paul Zimmet, Saravana Kumar, Gloria Mejia, Gary Wittert, Sara Jones, Jane Giles, Natalie Wischer, Sophia Zoungas, and Kim Morey received fund from National Health and Medical Research Council - Medical Research Future Fund (MRFF) Primary Health Care Research Initiative (PHCRI) [APP1200314]. The sponsor does not have any role in the study design, data collection and analysis, decision to publish, or preparation of the manuscript."  Please confirm that this does not alter your adherence to all PLOS ONE policies on sharing data and materials, by including the following statement: ""This does not alter our adherence to PLOS ONE policies on sharing data and materials.” (as detailed online in our guide for authors <http://journals.plos.org/plosone/s/competing-interests>).  If there are restrictions on sharing of data and/or materials, please state these. Please note that we cannot proceed with consideration of your article until this information has been declared.  Please include your updated Competing Interests statement in your cover letter; we will change the online submission form on your behalf. | Thank you, the competing interest section in the manuscript has now been amended to include “This does not alter our adherence to PLOS ONE policies on sharing data and materials.” as an adherence to PLOS ONE polices.  The cover letter has also been updated to include the competing interest section. | Page 27  Cover letter document |
| **3**. We note that you have indicated that there are restrictions to data sharing for this study. For studies involving human research participant data or other sensitive data, we encourage authors to share de-identified or anonymized data. However, when data cannot be publicly shared for ethical reasons, we allow authors to make their data sets available upon request. For information on unacceptable data access restrictions, please see <http://journals.plos.org/plosone/s/data-availability#loc-unacceptable-data-access-restrictions>.  Before we proceed with your manuscript, please address the following prompts:  a) If there are ethical or legal restrictions on sharing a de-identified data set, please explain them in detail (e.g., data contain potentially identifying or sensitive patient information, data are owned by a third-party organization, etc.) and who has imposed them (e.g., a Research Ethics Committee or Institutional Review Board, etc.). Please also provide contact information for a data access committee, ethics committee, or other institutional body to which data requests may be sent.  b) If there are no restrictions, please upload the minimal anonymized data set necessary to replicate your study findings to a stable, public repository and provide us with the relevant URLs, DOIs, or accession numbers. Please see <http://www.bmj.com/content/340/bmj.c181.long> for guidelines on how to de-identify and prepare clinical data for publication. For a list of recommended repositories, please see <https://journals.plos.org/plosone/s/recommended-repositories>. You also have the option of uploading the data as Supporting Information files, but we would recommend depositing data directly to a data repository if possible.  Please update your Data Availability statement in the submission form accordingly. | This manuscript is a study protocol with no previous pilot data; therefore, a dissemination plan of the results is included on page 25.  Data availability statement is provided with in the manuscript “This study applies the principles of Indigenous Data Sovereignty. Ethics approval and consent processes do not include approval for sharing the dataset. The study has ethics approval from SA Department of Health and Wellbeing Human Research Ethics Committee, approval number (2021/HRE00334) in April 2023. In addition, SA Aboriginal Health Research Ethics Committee (AHREC), approval number (04-21-969) in May 2022.”  The contact details:   - SA department for health and wellbeing research office: Phone: (08) 7117 6635 [HealthHumanResearchEthicsCommittee@sa.gov.au](mailto:HealthHumanResearchEthicsCommittee@sa.gov.au) - Aboriginal Health Council of South Australia Ltd   Tel: (08) 8273 7200  Email: [ahcsa@ahcsa.org.au](mailto:ahcsa@ahcsa.org.au) | Page 25  Page 26 |
| **Reviewer #1:** | | |
| As I understand it, the proposed trial design is summarised below. Right or wrong it would be very helpful in understanding this protocol if a schema of the design along the following lines is included in the Protocol itself. Period I Period II Stage 1 X 2 First Evaluation 3 4 Overall Evaluation Duration 6 months 10 weeks 6 months 10 weeks A PSN X PSN + E-L Washout PSN Randomisation B PSN X PSN Washout PSN+ E-L The above schema highlights one feature of the design that should be altered as randomisation should occur at the point when the intervention for the two groups diverges (marked at Stage X above).  Also, drawing randomisation from a hat is not regarded as an acceptable form of randomisation. An appropriate computer-based list should be drawn up by the statistical team which (perhaps) could be stratified by the size of the participating sites (the clusters).  Although this comment should be considered by the investigating team, I wonder whether the second period of the design is really worthwhile. Suppose, E-L is not an improvement over PSN alone in Period I, then there seems little point in carrying on into Period II. Obviously, if E-L does improve things then that might be different. So, my question is: Should the trial team consider establishing an independent Data Monitoring Committee to assess whether or not Period II should be implemented? This committee could be presented with a confidential analysis of the data from Period I to enable them to assess the situation and recommend any action (Note, this proposal is different from Data Monitoring as described on Page 21).  I too was concerned that the statistical analysis plan is over complex. Any analysis should be readily understandable to the non-statistical members of the investigating team and the readership of the eventual trial publication.  I rather suspect, for example, that on Page 12 “Analysis after the 12-month intervention follow-up is planned, spending alpha equal to 0.005”. will not be readily understood. Is it really necessary?  Summary This trial seems a very worthwhile project to undertake and the Protocol has been very carefully prepared. However, the design fault with respect to when, and how, randomisation is undertaken itself needs be altered. | Thank you for your valuable feedback. The research investigators have agreed that the randomisation can occur at six months. The manuscript text has been amended to include “Randomisation will be undertaken after six months of providing PSN for all enrolled health service sites.”  The manuscript text has been amended “All enrolled health service sites will be listed, and a statistician will be responsible for randomizing the health service sites into cluster one (Group A participants) or cluster two (Group B participants) using a block size through a computer program for randomisation.”  We would like to thank the reviewer for the comment. The training program was co-designed with seven Aboriginal health primary health services in South Australia, inclusive mainly of Aboriginal health practitioners and workers. It was imperative in the co-design process that all participants had the opportunity to do the training program. In the event that the hypothesis is verified as not effective for group A, a preliminary finding will be reported back to the project Steering Committee Members seeking a collaborative decision on the continuation of the training program for group B.  The statistical analysis plan has been reworded to include: A statistician, who is a Chief Investigator on the project has led the development of the statistical plan and will undertake the statistical analysis. A descriptive pre-post analysis of survey results due to participating in the PSN alone in the first six months will be calculated using appropriate parametric or non-parametric tests. These analyses will occur after each collection timepoint. In the case of participants who drop-out of the study, any data collected will be analysed by intention to treat. For the PSN, knowledge, attitude and practice of providers will be summarized using descriptive analyses. Differences between pre and post survey results will be calculated using appropriate parametric or non-parametric tests. Provision of evidence-based care and patient biomedical outcomes will be summarized using counts, means and percentages and differences between time collection points reported.  Directed Acyclic Graphs (DAGs) are being used to determine variables that need to be collected to adjust for potential confounding within statistical models. DAGs enable the conceptualisation of causal pathways. A DAG has been completed for each primary outcome. In addition, Generalized Linear Mixed-effects Models (GLMMs) are used to deal with outcomes with non-normal distributions, specifically to handle binary outcomes. GLMMs account for correlation structure within health care center (cluster) using a logit or log link for binary outcomes such as prevalence resulting in mixed effect logistic regression, while log link will be used for count outcomes resulting in mixed effects negative binomial regression. Effect measures will also be presented through calculation of number needed to treat and its associated 95% confidence interval.    This sentence has been deleted from the text “Analysis after the 12-month intervention follow-up is planned, spending alpha equal to 0.005”. | Page 10  Page 10  Pages 23-24 |
| **Reviewer #2:** | | |
| This study outlines a protocol for a Cluster Randomised Crossover Control Trial to be implemented in Aboriginal primary health care services in South Australia. The trial aims to assess the impact of a culturally and contextually tailored Aboriginal Diabetes Workforce Training Program on the knowledge, attitudes, confidence, skills, and practices of the Aboriginal primary health care workforce concerning diabetes care. The clarity of the study's objectives is commendable. The methodology is robust and appropriate, promising findings with practical implications. Consequently, publication in PLOS ONE is recommended. | Thank you for your feedback. |  |
| **Reviewer #3**: | | |
| There are some aspects of the study protocol which require further information in order to answer 'Yes' to the PLoS One reviewer questions. For instance, the protocol does not mention data availability once the study is complete (PLoS reviewer Q4). With regards to Q2 (meaningful outcome and testing of stated hypothesis) I have the following concerns:  1. **Sample size for training program.** Participation is voluntary and contact with the study team needs to be made by potential participants. The authors state there will be a minimum of 2 participants per site, from 10 potential Aboriginal Community Controlled Health Organization sites. Is there a strategy in case where there are fewer than 2 participants from a site, or several sites with no interested participants? It has been suggested the minimum sample size will be 20, but there is no guarantee that all sites will participate.  Can the authors indicate how many of the potential sites are urban, rural and remote as this is proposed comparison in the analysis? It is difficult to assess the applicability of the statistical methods without a sense of the expected sample size. | The manuscript text has been amended to include sample size calculation, drop-out rate and rationales:  **Sample size calculation and drop-out rate:**  The study population will be drawn from primary health settings and will include Aboriginal Health Workers and Practitioners, and multidisciplinary health care providers across South Australia. Based on our scoping review “Supporting best practice in the management of chronic diseases in primary health care settings: a scoping review of training programs for Indigenous Health Workers and Practitioners” currently under review, the review reported improvement in participants’ knowledge and confidence of a similar population group between 12.7%-20.97% increase pre-post training, and an increase in confidence in both clinical and non-clinical skills. The sample size for the studies included in this review ranged from 4 to 250 with a total of 1120 participants. The drop-out rate was reported to be between 40 to 70% during the follow up period. Furthermore, based on the publicly available information from the Australian Health Practitioner Regulation Agency (AHPRA), a total of (n=100) Aboriginal health practitioners are currently registered and licenced to work in health settings across South Australia (This does not include Aboriginal Health Workers). There are 11 Aboriginal community-controlled health services and 10 South Australian Local Health Networks, some of which have expressed interest in participating in the study. Overall, we have estimated an uptake of (n=40) and accounted for drop-out rate of 50% of the AHW/P, therefore, (n=20) participants would need to complete the training. This number aligns with previous studies (14-16).  As participation in this project is voluntary, potential participating sites may decline to participate and participants may withdraw from the study. In the event that a participant wishes to withdraw from the study, they will be asked to complete a participant withdrawal form, and this will be reported in the final findings. A minimum of two participants will be recruited from each study site, with no restriction on the maximum number of participants from each service. Recruitment will include participants from different primary health service settings, including urban, rural, and very remote sites. We aim to include a minimum of two sites in each setting. Three months has been allocated to recruit participants.  The manuscript text has been amended to include “Recruitment will include participants from primary health service settings, across urban, regional, and remote locations. We aim to include a minimum of two sites in each setting.” | Pages (8-9)  Page 9 |
| **2. Patient outcomes.** It appears that medical records for all diabetes patients at each participating health service will be reviewed, but a yet-to-be determined proportion of practitioners will participate in the training. Will the researchers be able to link patients to specific health practitioners to look for changes in practice? If not, the authors should discuss how likely it is they will see an effect of the intervention if only a small number of practitioners at a site participate. | In addition to the responses to the previous question related to sample size.  Patient outcomes data and training participant data will not be linked. In the health services where the majority of the eligible workforce participated in the training program, it would be likely to expect changes within these services and an improvement in the quality of care provided at the population level. While, sites with a small proportion of their workforce participating, it would be unlikely to expect change at a population level in diabetes management. In both scenarios, the direct impact of the training program on patient outcomes related to diabetes management and the quality of care will not be clearly established. The review of patient outcomes was a request by some services in the co-design of the program. | Page 21 |
| 3. There is no mention of drop-out from the study. How will data for participants who do not complete the online training be treated?  Is there a minimum number of PSN sessions a participant needs to attend to be considered as an 'active' participant? | The manuscript text was amended to include the drop-out rate with the sample size calculation.  In regard to the drop-out rate and the data for participants, the manuscript has been amended to include “In the case of participants who drop-out of the study, any data collected will be analysed by intention to treat.”  As there is limited evidence on the relationship between participation rate in networks and effect on learning outcomes, we will apply an arbitrary percentage. The minimum participation rate in in PSN sessions in order to be considered as an active participant will be 50% of all the total sessions. | Page 8  Page 23  Page 24 |
| 4. The description of the statistical analysis could be more specific about the hypotheses being tested. For instance, the GLMM is proposed for the 12-month post-intervention analysis, but it is not clear what the hypothesis is. Pre-post analysis of PSN survey results is mentioned using parametric or non-parametric tests. Is there any reason why the GLMM could not be used so the clustering effect is accounted for? I would also expect the knowledge, skills and confidence for an individual may fluctuate over time. How is this being addressed, or are the researchers only interested in comparing results at two specific time points in any one analysis? | Thank you for your feedback. The quantitative analysis section was rewritten. Please check response provided to a comment from reviewer 1. | Pages 23-24 |
| **Other comments:** 5. Abstract and discussion says a final report will be produced in 2025. I am not sure how this can occur when the study will be conducted between Feb 2024 and September 2026, with T6 29 months post-baseline. | Thank you, this was amended in the manuscript | Pages 4 and 24 |
| 6. Training program eligibility. It appears that practitioners who have a Graduate Certificate of Diabetes Education and Management are eligible for the intervention. The authors could discuss how inclusion of participants with a higher starting level of knowledge may impact results. | Completion of certificate 3 or 4 is the minimum requirement for Aboriginal Health Workers (Cert III) and Practitioners (cert IV) to be enrolled in the training program. These are the minimum qualifications for the respective professions to practice in South Australia.  Furthermore, the manuscript has been amended to include “Inclusion of participants with a higher starting level of knowledge in the training program could certainly influence the results in various ways; participants with a higher baseline knowledge could affect the overall evaluation of the project, as they might show less improvement than their peers throughout the training program. However, this could have a positive effect for peer support network and onsite practice support, as they will share their experiences and knowledge with their peers. This could lead to skewed perception of the interventions’ effectiveness. The main aim is to recruit all interested participants who have completed or currently enrolled in certificate III/IV in Indigenous primary health care, and there will be individual evaluation for all participants to complete at certain timepoints to establish baseline status.” | Pages 22-23 |
| 7. Eligibility for interviews about enablers and barriers. Have the authors considered the potential bias introduced if only participants who complete the training are eligible for interview? Participants who do not complete the training may present a unique set of barriers not captured by those who complete. | Thank you for this suggestion. The manuscript has been amended to include:  “All training program participants are eligible for an interview on the enablers and barriers to participation, effectiveness and sustainability of the training program. These interviews are one component of the training program evaluation. The overall evaluation will seek to understand the effectiveness of the training program on knowledge, confidence, attitude, skills, and practice of Aboriginal Primary Health Care Service providers in providing care to clients with diabetes. However, including only participants who completed the training program for the interview, might introduce bias, therefore, a sensitivity test could be undertaken. As part of this, the research team will invite all participants who have completed or did not complete the training program for an interview.” | Page 23 |
| 8. Top of page 14. T2, T4 and T6 are used prior to their explanation. | Thank you, this was amended in the manuscript for clarity | Page 14 |
| 9. Page 14. “The secondary outcomes for this study relate to the patients care received and patients’ clinical outcomes”. However, the first three dot points that follow focus on the practitioner or health service, not the patient. | Thank you, this was amended in the manuscript for clarity | Page 15 |
| 10. Page 21. What potential serious adverse events do the authors envisage could develop? | In addition to safety consideration section on page 21-22, the adverse event section within the manuscript has been amended to include “It is anticipated that the risks associated with participating in this research project will be minimal and limited to discomfort or inconvenience.” | Page 22 |
| **Reviewer #4:** | | |
| It is a well written protocol. Minor language editing is required. A clarification on the working conditions are required. Will the health workers or service providers be able to provide the services after training? It will be good if you give some clarification on why and how trainings will help the staff to address issues of diabetic patients. Diabetes requires self-care by the patients and family members. Though self-management is mentioned but would be good if you add a module on training staff on how to train patients on tailored self-care. Best wishes | Thank you for your feedback. |  |
