# Supplementary material for: Translation of culturally and contextually informed diabetes training for Aboriginal primary health care providers on Aboriginal client outcomes: Protocol of a cluster randomized crossover trial of effectiveness
Source: PLoS One. 2024 Jul 23;19(7):e0305472. doi: 10.1371/journal.pone.0305472 (PMC11265707; doi:10.1371/journal.pone.0305472)
Supplement: Attachment — Submitted filename: Authors response to reviewers feedback 30-4-2024.docx [file pone.0305472.s011.docx]

| **Feedback items** | **Authors response (Amended/added)** | **Page and line number** |
| --- | --- | --- |
| **Journal requirements** | | |
| Please review your reference list to ensure that it is complete and correct. If you have cited papers that have been retracted, please include the rationale for doing so in the manuscript text, or remove these references and replace them with relevant current references. Any changes to the reference list should be mentioned in the rebuttal letter that accompanies your revised manuscript. If you need to cite a retracted article, indicate the article’s retracted status in the References list and also include a citation and full reference for the retraction notice. | Thank you for your feedback on the reference lists.  All references have been cross-checked and amended when required.  At the introduction section: the first paragraph has been updated with in text citation.  Reference (2): “Intensive blood-glucose control with sulphonylureas or insulin compared with conventional treatment and risk of complications in patients with type 2 diabetes (UKPDS 33). The Lancet (British edition). 1998;352(9131):837-53.”, was replaced with “Stratton IM, Adler AI, Neil HA, Matthews DR, Manley SE, Cull CA, et al. Association of glycaemia with macrovascular and microvascular complications of type 2 diabetes (UKPDS 35): prospective observational study. Bmj. 2000;321(7258):405-12.”  Reference (5): Australian Institute of Health and Welfare. The Health and Welfare of Australia’s Aboriginal and Torres Strait Islander Peoples: 2008. Canberra: AIHW; 2008. This reference was replaced with “Australian Institute of Health and Welfare. Aboriginal and Torres Strait Islander Health Performance Framework - Summary report March 2024. AIHW; 2024.”  Reference (10): “Gibson, O., The impact of primary health care on hospitalisation of Aboriginal and Torres Strait Islander adults with type 2 diabetes in far north Queensland, Australia. 2013, University of South Australia,: Adelaide.” This reference was replaced with “Thomas SL, Zhao Y, Guthridge SL, Wakerman J. The cost-effectiveness of primary care for Indigenous Australians with diabetes living in remote Northern Territory communities. Medical Journal of Australia. 2014;200(11):658-62.”  Reference (23), was same as reference (15), therefore, it was deleted as a duplicate. “Colleran K, Harding E, Kipp BJ, Zurawski A, MacMillan B, Jelinkova L, et al. Building Capacity to Reduce Disparities in Diabetes: Training Community Health Workers Using an Integrated Distance Learning Model. The Diabetes educator. 2012;38(3):386-96.”  Reference (25) “Australian Government. Good Clinical Practice (GCP) in Australia. In: The Commonwealth Department of Health and Aged Care, editor. 2020”  This reference was replaced with “Australian Government. Good Clinical Practice (GCP) inspection program Guidance for GCP inspection of clinical trial sites for investigational biologicals and medicinal products. In: The Commonwealth Department of Health and Aged Care, editor. 2022.” | Page 4  Pages 27-28 |
| **Reviewer #4:** | | |
| 1- Minor language editing is required. I would strongly suggest language. example for that is in Abstract as well. The last sentence .... health workers outcome? Is it health workers outcome or performance? Outcomes are not the same as performance and misleads the reader. I would suggest the authors to address such issues carefully. 2- A clarification on the working conditions are required. Will the health workers or service providers be able to provide the services after training? It will be good if you give some clarification on why and how trainings will help the staff to address issues of diabetic patients. 3- Diabetes requires self-care by the patients and family members. Though self-management is mentioned but would be good if you add a module on training staff on how to train patients on tailored self-care. | 1- Thank you for your feedback. Health workers outcomes was replaced with “ …health care provider knowledge, attitude, confidence, skill and practice relating to diabetes care”.   In regards to health care providers outcomes: We are not measuring AHW/Ps performance, we are evaluating the effectiveness of the training program on Aboriginal Health Workers, Practitioners and multidisciplinary team knowledge, confidence, skill, practice and attitude related to diabetes care, and secondary outcomes relating to quality of diabetes care provided and patient outcomes.   2- There will be a follow-up for 12 months following the training program, however, we cannot guarantee that they will continue providing these services after the program finishes. In addition, before the training program finishes, we will follow up with the services and discuss their feedback, and check if they require further workshops/training.   **Why/how the training will help the staff to address issues of diabetes patients?**  Prevalence of self-reported type 2 diabetes among adults Aboriginal and Torres Strait Islander people is estimated of 10.7% in 2018-19 across Australia. It is therefore, expected that about 10.7% of the clients’ medical data at each health service will be included in the medical record review. Therefore, equipping the AHW/Ps with the training program, will help to enhance the quality of care provided to diabetes patients which will leads to improving patients’ health outcome and quality of care at each health service. However, in the health services where the majority of the eligible workforce participated in the training program, it would be likely to expect changes within these services and an improvement in the quality of care provided at the population level. While, sites with a small proportion of their workforce participating, it would be unlikely to expect change at a population level in diabetes management. In both scenarios, the direct impact of the training program on patient outcomes related to diabetes management and the quality of care will not be clearly established. The review of patient outcomes was a request by some services in the co-design of the program.  The training program has three components (Peer Support Network, E-learning modules and On-Site Support), these components will be accompanied by Systems assessment and Medical record review.  Upon completion of the training program, an evaluation will be conducted for the participating Aboriginal Health Workers (AHW/Ps). The results of this evaluation will be shared with the Aboriginal primary care health services.  During the monthly session of the Peer Support Network, the aggregated results of the survey evaluation will be presented to the service. These results will serve as a valuable resource for enhancing the effectiveness of the Network.  For the On-site practice support, participants will be given an opportunity to implement knowledge into practice within their primary health care settings.  In terms of systems assessment, each health service will receive their individual assessment within three months of its completion. As part of the medical record review each health service will be provided with aggregated and analyzed data specific to their service level. All participating services in the study will receive combined aggregated results of the audits as they become available These aggregated data will help to improve the health service and the participants outcomes/performance on managing diabetes. This comprehensive approach ensures continuous improvement and promotes participants’ attitude toward diabetes management and will provide insight into the effectiveness of the training program on patients’ outcomes. It empowers health services and their health care staff to achieve better health outcomes for their clients. The overall objective of the training program is to evaluate the effectiveness of the training program on Aboriginal Health Workers, Practitioners and multidisciplinary team knowledge, confidence, skill, practice and attitude related to diabetes care, and secondary outcomes relating to quality of diabetes care provided and patient outcomes.   3- Thank you for your valuable feedback. Yes, the e-learning modules are designed to equip the AHW/Ps with the essential knowledge and skills to support the diabetic patients on their self-management skills. The modules will cover a wide range of topics starting from the very basics, moving on to complex aspects of diabetes management. The modules start with a discussion of signs and symptoms, healthy living and ways of diabetes prevention, moving through to learning of Glucose monitoring, how to identify low and high blood glucose levels, and managing medicines and insulins. A module on the diabetes related complications such as Kidney disease, eye disease and foot complications. This module will be followed by support for self-management, where participants learn how to support diabetic patients on managing their diabetes on a daily basis. In addition, participants will acquire more knowledge and understanding on managing diabetes for high risk groups such as pregnancy, elderly and children. Overall, by completing the E-learning Modules participants will be able to support diabetic patients on how to self-manage their diabetes. | Page 4 |
